# Optimal and Numerical Solutions for an MHD Micropolar Nanofluid between Rotating Horizontal Parallel Plates

**DOI:** 10.1371/journal.pone.0124016

**Published:** 2015-06-05

**Authors:** Sohail Nadeem, Sadaf Masood, Rashid Mehmood, Muhammad Adil Sadiq

**Affiliations:** 1 Department of Mathematics, Quaid-i-Azam University, 45320, Islamabad, 44000, Pakistan; 2 Dammam Community College, Department of Mathematics, KFUPM, Dharan, 31261, Saudi Arabia; North China Electric Power University, CHINA

## Abstract

The present analysis deals with flow and heat transfer aspects of a micropolar nanofluid between two horizontal parallel plates in a rotating system. The governing partial differential equations for momentum, energy, micro rotation and nano-particles concentration are presented. Similarity transformations are utilized to convert the system of partial differential equations into system of ordinary differential equations. The reduced equations are solved analytically with the help of optimal homotopy analysis method (OHAM). Analytical solutions for velocity, temperature, micro-rotation and concentration profiles are expressed graphically against various emerging physical parameters. Physical quantities of interest such as skin friction co-efficient, local heat and local mass fluxes are also computed both analytically and numerically through mid-point integration scheme. It is found that both the solutions are in excellent agreement. Local skin friction coefficient is found to be higher for the case of strong concentration i.e. n=0, as compared to the case of weak concentration n=0.50. Influence of strong and weak concentration on Nusselt and Sherwood number appear to be similar in a quantitative sense.

## Introduction

The idea of micropolar fluid was introduced by Eringen [1–2]. This idea is a substantial generalization of the classical Navier Stokes model to discuss certain complex fluids. This particular class of fluids consists of rigid, randomly oriented spherical particles with microstructures such as liquid crystals, colloidal fluids, polymeric suspensions, hematological suspensions and animal blood etc. Extensive uses of micropolar fluid theory are given in the books of Lukaszewicz [3] and Eringen [4]. Bhargava et al [5] presented finite element solutions for mixed convective micropolar flow driven by a porous stretching sheet. Later on, Takhar et al [6] highlighted free convection MHD micropolar fluid flow between two porous vertical plates and perceived that velocity decreases with an increase in Hartman number. Stagnation point flow of a micropolar fluid towards a stretching sheet was investigated by Nazar et al [7]. Ziabakhsh et al [8] presented Homotopy analysis solutions of micropolar flow in a porous channel with heat and mass transfer. Similarly, Ishak et al [9] considered magnetohydrodynamic flow of micropolar fluid towards a stagnation point on a vertical surface. Joneidi et al [10] inspected behavior of micropolar flow in a porous channel with high mass transfer. Influence of chemical reaction and thermal radiation on MHD micropolar flow over a vertical moving porous plate in a porous medium with heat generation was discussed by Mohamed et al [11] who found that the translational velocity across the boundary layer and the magnitude of micro rotation at the wall decreased with an increase in magnetic field and Prandtl number. Some noteworthy studies on micropolar fluids with certain physical constraints can be found in [12–16].

In the last few decades nanofluids have proved to be extremely promising heat transfer agents in modern day industry and in numerous engineering applications of global interest like biomedical, optical and electronic fields. Choi [17] initiated the astonishing idea of nanofluids. Later numerous researchers and scientists investigated various real life flow problems under the influence of nanofluids. Buongiorno [18] presented a novel study on describing flow and heat transfer mechanisms of nanofluids. Similarly Nield et al [19] discussed natural convective boundary-layer flow of a nanofluid past a vertical plate and observed that the reduced Nusselt number is a decreasing function of thermophoresis and Brownian motion parameter. Nadeem et al [20] presented the optimized analytical solution for oblique flow of a Casson nanofluid with convective boundary conditions. They found that nanoparticle concentration is an increasing function of stretching parameter and Brownian motion while it is a decreasing function of thermophoresis, Biot number and non-Newtonian (Casson) parameter. Ganji et al [21] conducted a valuable study on heat transfer of Cu-Water nanofluid flow between parallel plates and concluded that heat flux at the surface has a direct relationship with nanoparticle volume fraction. Ganji et al [22] discussed simulation of MHD Cuo-Water nanofluid flow and convective heat transfer under the influence of Lorentz forces. Some notable studies on the topic can be found in [23–41].

The main goal of the present study is to discuss hydromagnetic flow of a micropolar nanofluid between parallel plates. Optimal homotopy analysis method proposed by Liao [24–25] is utilized to obtain graphical results against embedding physical parameters. Numerical values for skin friction, Nusselt and Sherwood numbers are obtained through OHAM and also numerically through mid-point integration scheme [26–27]. It would be obligatory to mention that mid-point integration scheme is used to verify our obtained analytical results. It is found that both the results are in a very good agreement with each other.

## Mathematical Formulation

**Fig 1:**
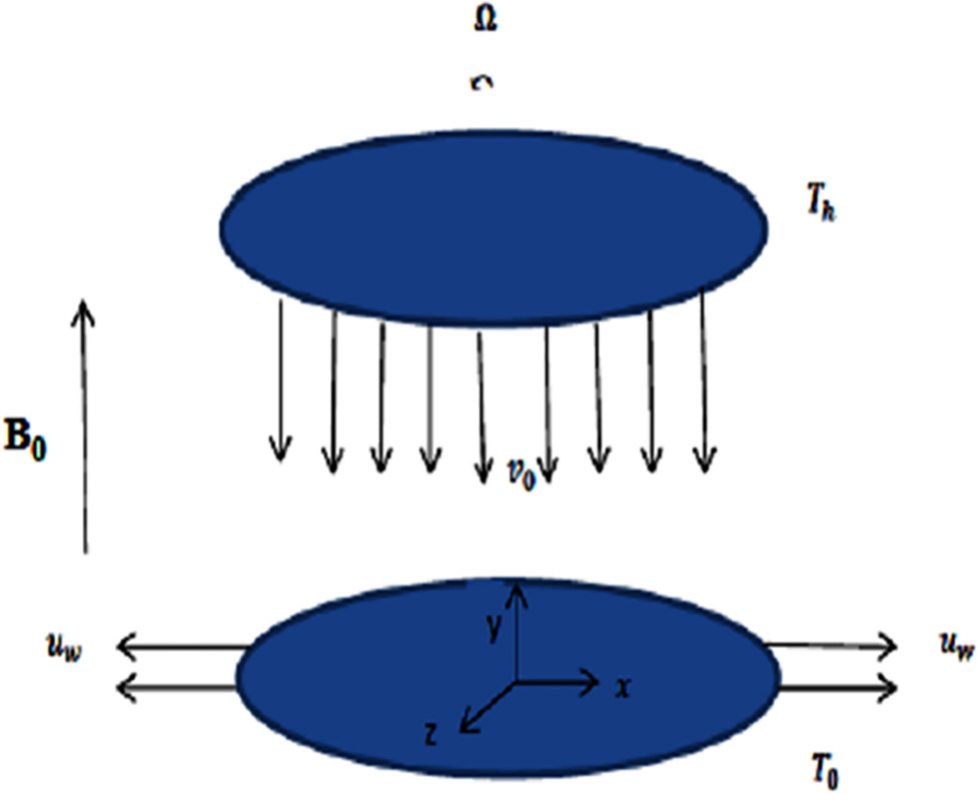
Physical description of the problem.

Consider steady incompressible 3D flow of an electrically conducting micropolar nanofluid between two horizontal parallel plates. Both the fluid and the plates rotate together around the *y-axis* with a constant angular velocity *Ω*. The *x* and *y* − axes are taken along and perpendicular to the plate respectively while the *z* − axis is taken normal to the *xy* plane. The plates are placed at *y* = 0 and *y* = *h*. The lower plate is being stretched by two equal and opposite forces so that the position of the point (0,0,0) remains unchanged. A uniform magnetic flux with density *B*_0_ acts along the y-axis. The upper plate is subject to constant wall suction velocity *v*_0_(< 0) or constant wall injection velocity *v*_0_(> 0) respectively as shown in Fig 1.

The governing equations of the flow problem can be stated as [5–11, 18]

$$\frac{\partial u}{\partial x}+\frac{\partial v}{\partial y}=0,$$ (1)

$$u\frac{\partial u}{\partial x}+v\frac{\partial u}{\partial y}+2\Omega w=-\frac{1}{\rho }\frac{\partial p}{\partial x}+\left(\nu +\frac{k_{1}^{*}}{\rho }\right)\nabla^{2}u+\frac{k_{1}^{*}}{\rho }\frac{\partial N}{\partial y}-\frac{\sigma }{\rho }B_{0}^{2}u,$$ (2)

$$u\frac{\partial v}{\partial x}+v\frac{\partial v}{\partial y}=-\frac{1}{\rho }\frac{\partial p}{\partial y}+\left(\nu +\frac{k_{1}^{*}}{\rho }\right)\nabla^{2}v+\frac{k_{1}^{*}}{\rho }\frac{\partial N}{\partial x},$$ (3)

$$u\frac{\partial w}{\partial x}+v\frac{\partial w}{\partial y}-2\Omega u=\left(\nu +\frac{k_{1}^{*}}{\rho }\right)\nabla^{2}w-\frac{\sigma }{\rho }B_{0}^{2}w,$$ (4)

$$u\frac{\partial T}{\partial x}+v\frac{\partial T}{\partial y}=\frac{k}{\rho C_{p}}\nabla^{2}T+\tau \left(D_{B}\nabla C.\nabla T+\frac{D_{T}}{T_{\infty }}\nabla T.\nabla T\right),$$ (5)

$$u\frac{\partial N}{\partial x}+v\frac{\partial N}{\partial y}=\frac{\gamma }{\rho j}\nabla^{2}N-\frac{2k_{1}^{*}}{\rho j}N+\frac{k_{1}^{*}}{\rho j}\left(\frac{\partial v}{\partial x}-\frac{\partial u}{\partial y}\right),$$ (6)

$$u\frac{\partial C}{\partial x}+v\frac{\partial C}{\partial y}=D_{B}\nabla^{2}C+\frac{D_{T}}{T_{\infty }}\nabla^{2}T.$$ (7)

The appropriate boundary conditions are

$$\begin{array}{c} u=ax,\;v=0,w=0,\;T=T_{0},N=-n\frac{\partial u}{\partial y},\;C=C_{0}\;at\;y=0,\\ u=0,v=v_{0},w=0,T=T_{h},N=n\frac{\partial u}{\partial y},\;C=C_{h}\;at\;y=h, \end{array}$$ (8)

where *u*, *v* and *w* are the velocity components along the *x*, *y*, *z* — directions respectively while *u* = *ax* shows that lower plate is stretching.*ρ* is the density, *ϑ* is the kinematic viscosity, $k_{1}^{*}$ is the vortex viscosity, *N* is the micro rotation velocity, *Ω* is the rotation velocity, *B*_0_ is the magnetic field, *σ* is electrical conductivity, *α* is thermal diffusivity, *k* is the thermal conductivity, *C*_*p*_ is the specific heat of the fluid, *j* is the micro-inertia density, *v*_0_ is the suction/injection velocity, *T* is the temperature of the fluid, *T*_0_ is the temperature at lower plate, *T*_*h*_ is the temperature at upper plate. *D*_*B*_ Brownian diffusion coefficient, *D*_*T*_ is the thermophoresis diffusion coefficient, *C* is the concentration and *n* is the boundary parameter. The case *n* = 0 represents strong concentration, *n* = 0.5 indicates weak concentration and vanishing of the antisymmetric part of the stress tensor and *n* = 1 represents turbulent flow. Spin gradient viscosity *γ* is defined as $\gamma =\left(\mu +\frac{k_{1}^{*}}{2}\right)j.$

Using transformations

$$\begin{array}{r} u=axf\prime \left(\eta \right),\;v=-ahf\left(\eta \right),\;w=axg\left(\eta \right),\;\\ N=\frac{axG\left(\eta \right)}{h},\theta \left(\eta \right)=\frac{T-T_{h}}{T_{0}-T_{h}},\;\varphi \left(\eta \right)=\frac{C-C_{h}}{C_{0}-C_{h}},\;\eta =\frac{y}{h} \end{array}\}.$$ (9)

Eq (1) is identically satisfied while Eqs. (2)–(7), in dimensionless form are given by

$$\left(1+N_{1}\right)f^{iv}-R\left(f\prime f\prime \prime -ff\prime \prime \prime \right)+N_{1}G\prime \prime -Mf\prime \prime -2Krg\prime =0,$$ (10)

$$\left(1+N_{1}\right)g\prime \prime +R\left(fg\prime -gf\prime \right)-Mg+2Krf\prime =0,$$ (11)

$$\theta \prime \prime +Pr\left(Rf\theta \prime +N_{b}\theta \prime \phi \prime +N_{t}{\theta \prime }^{2}\right)=0,$$ (12)

$$N_{2}G\prime \prime -N_{1}\left(2G+f\prime \prime \right)-N_{3}R\left(f\prime G-G\prime f\right)=0,$$ (13)

$$\phi \prime \prime +R.Scf\phi \prime +\frac{N_{t}}{N_{b}}\theta \prime \prime =0,$$ (14)

with boundary conditions

$$\begin{array}{c} f\left(0\right)=0,\;f\prime \left(0\right)=1,\;g\left(0\right)=0,\;\theta \left(0\right)=1,G\left(0\right)=-nf\prime \prime \left(0\right),\phi \left(0\right)=1,\\ f\left(1\right)=\lambda ,f\prime \left(1\right)=0,\;g\left(1\right)=0,\;\theta \left(1\right)=0,G\left(1\right)=nf\prime \prime \left(1\right),\;\phi \left(\infty \right)=0, \end{array}$$ (15)

where primes denote differentiation with respect to *η*. *N*_1_ is the coupling parameter, *N*_2_ is the spin-gradient viscosity parameter, *R* is the Reynold number, *M* is the magnetic parameter, *Kr* is the rotation parameter, *Pr* is the Prandtl number, *Sc* is the Schmidt number, *N*_*b*_ is the Brownian motion parameter and *N*_*t*_ is the thermophoresis parameter which are defined by

$$\begin{array}{l} N_{1}=\frac{k_{1}^{*}}{\mu },N_{2}=\frac{\nu_{s}}{\nu h^{2}},N_{3}=\frac{j}{h^{2}},R=\frac{ah^{2}}{\nu },M=\frac{\sigma h^{2}B_{0}^{2}}{\rho \nu },Kr=\frac{h^{2}\Omega }{\nu },Pr=\frac{\mu C_{p}}{k},\\ \tau =\frac{{\left(\rho C_{p}\right)}_{nf}}{{\left(\rho C_{p}\right)}_{f}},N_{t}=\frac{\tau D_{T}(T_{0}-T_{h})}{\nu T_{\infty }},N_{b}=\frac{\tau D_{B}(C_{0}-C_{h})}{\nu },Sc=\frac{\nu }{D_{B}},\lambda =-\frac{\nu_{0}}{h}. \end{array}$$ (16)

The skin friction coefficient *C*_*f*_, Nusselt number *Nu* and Sherwood number *Sh* are defined as

$$C_{f}=\frac{\tau_{w}}{\rho u_{w}^{2}},Nu=\frac{hq_{w}}{k{(T}_{0}-T_{h})},Sh=\frac{hj_{w}}{D_{B}{(C}_{0}-C_{h})},$$ (17)

where

$$\tau_{w}={(\left(\mu +k_{1}^{*}\right)\frac{\partial u}{\partial y}+k_{1}^{*}N)}_{y=0},q_{w}=-k{\left(\frac{\partial T}{\partial y}\right)}_{y=0},\;j_{w}=-D_{B}{\left(\frac{\partial C}{\partial y}\right)}_{y=0}.$$ (18)

The dimensionless forms of skin friction coefficient Cf^, Nusselt number *Nu* and Sherwood number *Sh* are

Cf^=(1+N1)f″(0)+N1G(0),Nu=−θ′(0),Sh=−ϕ′(0). (19)

## Method of Solution

### 1. Optimal HAM Solution

The governing system of coupled ordinary differential Eqs (10)–(14) is nonlinear and extremely complicated in nature. These equations are solved analytically by optimal homotopy analysis method (OHAM). Following Liao [24–25] we know that *f*(*η*), *g*(*η*), *θ*(*η*), *G*(*η*) and *ϕ*(*η*) can be expressed by a set of a certain type of exponential base functions

$$\left\{\eta^{k}\;\text{exp}\left(-m\eta \right)\;|k\geq 0,\;m\geq 0\right\},$$ (20)

in the forms

$$f\left(\eta \right)=a_{0,0}^{0}+\sum_{m=0}^{\infty }\sum_{k=0}^{\infty }a_{n,m}^{k}y^{k}\;\text{exp}\left(-my\right),$$ (21)

$$g\left(\eta \right)=\sum_{m=0}^{\infty }\sum_{k=0}^{\infty }b_{n,m}^{k}y^{k}\;\text{exp}\left(-my\right),$$ (22)

$$\theta \left(\eta \right)=\sum_{m=0}^{\infty }\sum_{k=0}^{\infty }c_{n,m}^{k}y^{k}\;\text{exp}\left(-my\right),$$ (23)

$$G\left(\eta \right)=\sum_{m=0}^{\infty }\sum_{k=0}^{\infty }d_{n,m}^{k}y^{k}\;\text{exp}\left(-my\right),$$ (24)

$$\phi \left(\eta \right)=\sum_{m=0}^{\infty }\sum_{k=0}^{\infty }e_{n,m}^{k}y^{k}\;\text{exp}\left(-my\right),$$ (25)

in which $a_{m,n}^{k},\;b_{m,n}^{k},\;c_{m,n}^{k},d_{m,n}^{k}$ and $e_{m,n}^{k}$ are series coefficients. The initial guesses *f*_0_, *g*_0_, *θ*_0_, *G*_0_ and *ϕ*_0_ for *f*(*η*), *g*(*η*), *θ*(*η*), *G*(*η*) and *ϕ*(*η*) are selected as follows

$$f_{0}\left(\eta \right)=\left(1-2\lambda \right)\eta^{3}+(3-2\lambda )\eta^{2}+\eta ,$$ (26)

$$g_{0}\left(\eta \right)=0,$$ (27)

$$\theta_{0}\left(\eta \right)=1-\eta ,$$ (28)

$$G_{0}\left(\eta \right)=-n\left(6\lambda -4+2\eta \right),$$ (29)

$$\phi_{0}\left(\eta \right)=1-\eta .$$ (30)

We select the auxiliary linear operators as

$$L_{f}=\frac{d^{4}f}{d\eta^{4}},L_{g}=\frac{d^{2}g}{d\eta^{2}},L_{\theta }=\frac{d^{2}\theta }{d\eta^{2}},L_{G}=\frac{d^{2}G}{d\eta^{2}},L_{\phi }=\frac{d^{2}\phi }{d\eta^{2}}\}.$$ (31)

The above linear operators have the following properties

$$L_{f}\left\{C_{0}+C_{1}\eta +C_{2}\eta^{2}+C_{3}\eta^{3}\right\}=0,$$ (32)

$$L_{g}\left\{C_{4}+C_{5}\eta \right\}=0,$$ (33)

$$L_{\theta }\left\{C_{6}+C_{7}\eta \right\}=0,$$ (34)

$$L_{G}\left\{C_{8}+C_{9}\eta \right\}=0,$$ (35)

$$L_{\phi }\left\{C_{10}+C_{11}\eta \right\}=0,$$ (36)

Where *C*_0_ − *C*_11_ are arbitrary constants.

The zeroth order homotopic deformation equations can be written as

$$\left(1-p\right)L_{f}\left\{\hat{f}\left(\eta ;p\right)-f_{0}\left(\eta \right)\right\}=pc_{0}^{f}N_{f}\left(\hat{f}\left(\eta ;p\right),\hat{g}\left(\eta ;p\right),\hat{G}\left(\eta ;p\right)\right),$$ (37)

$$\left(1-p\right)L_{g}\left\{\hat{g}\left(\eta ;p\right)-g_{0}\left(\eta \right)\right\}=pc_{0}^{g}N_{g}\left(\hat{f}\left(\eta ;p\right),\hat{g}\left(\eta ;p\right)\right),$$ (38)

$$\left(1-p\right)L_{\theta }\left\{\hat{\theta }\left(\eta ;p\right)-\theta_{0}\left(\eta \right)\right\}=pc_{0}^{\theta }N_{\theta }\left(\hat{f}\left(\eta ;p\right),\hat{\theta }\left(\eta ;p\right),\hat{\phi }\left(\eta ;p\right)\right),$$ (39)

$$\left(1-p\right)L_{G}\left\{\hat{G}\left(\eta ;p\right)-G_{0}\left(\eta \right)\right\}=pc_{0}^{G}N_{G}\left(\hat{f}\left(\eta ;p\right),\hat{G}\left(\eta ;p\right)\right),$$ (40)

$$\left(1-p\right)L_{\phi }\left\{\hat{\phi }\left(\eta ;p\right)-\phi_{0}\left(\eta \right)\right\}=pc_{0}^{\phi }N_{\phi }\left(\hat{f}\left(\eta ;p\right),\hat{\theta }\left(\eta ;p\right),\hat{\phi }\left(\eta ;p\right)\right),$$ (41)

$$\hat{f}\left(0;p\right)=0,\hat{f\prime }\left(0;p\right)=0,\hat{g}\left(0;p\right)=0,\hat{\theta }\left(0;p\right)=0,\hat{G}\left(0;p\right)=-n\hat{f\prime \prime }\left(0;p\right),\hat{\phi }\left(0;p\right)=0,$$ (42)

$$\hat{f}\left(1;p\right)=0,\hat{f\prime }\left(1;p\right)=0,\hat{g}\left(1;p\right)=0,\hat{\theta }\left(1;p\right)=0,\hat{G}\left(0;p\right)=n\hat{f\prime \prime }\left(0;p\right),\hat{\phi }\left(1;p\right)=0,$$ (43)

where *p ϵ* [0, 1] indicates the embedding parameter and $c_{0}^{f},\;c_{0}^{g},\;c_{0}^{\theta },c_{0}^{G}$ and $c_{0}^{\phi }$ the nonzero auxiliary parameters. Moreover the nonlinear operators *N*_*f*_, *N*_*g*_, *N*_*θ*_, *N*_*G*_ and *N*_*ϕ*_ are prescribed as

$$N_{f}\left(\hat{f}\left(\eta ;p\right),\hat{g}\left(\eta ;p\right),\hat{G}\left(\eta ;p\right)\right)=\left(1+N_{1}\right)\frac{\partial^{4}\hat{f}\left(\eta ;p\right)}{\partial \eta^{4}}-R\left(\frac{\partial \hat{f}\left(\eta ;p\right)}{\partial \eta }\frac{\partial^{2}\hat{f}\left(\eta ;p\right)}{\partial \eta^{2}}-\hat{f}\left(\eta ;p\right)\frac{\partial^{3}\hat{f}\left(\eta ;p\right)}{\partial \eta^{3}}\right)+N_{1}\frac{\partial^{2}\hat{G}\left(\eta ;p\right)}{\partial \eta^{2}}-M\frac{\partial^{2}\hat{f}\left(\eta ;p\right)}{\partial \eta^{2}}-2Kr\frac{\partial \hat{g}\left(\eta ;p\right)}{\partial \eta },$$ (44)

$$N_{g}\left(\hat{f}\left(\eta ;p\right),\hat{g}\left(\eta ;p\right)\right)=\left(1+N_{1}\right)\frac{\partial^{2}\hat{g}\left(y;p\right)}{\partial \eta^{2}}+R\left(\hat{f}\left(\eta ;p\right)\frac{\partial \hat{g}\left(\eta ;p\right)}{\partial \eta }-\hat{g}\left(\eta ;p\right)\frac{\partial \hat{f}\left(\eta ;p\right)}{\partial \eta }\right)-M\hat{g}\left(\eta ;p\right)+2Kr\frac{\partial \hat{f}\left(\eta ;p\right)}{\partial \eta },$$ (45)

$$N_{\theta }\left(\hat{f}\left(\eta ;p\right),\hat{\theta }\left(\eta ;p\right),\hat{\phi }\left(\eta ;p\right)\right)=\frac{\partial^{2}\hat{\theta }\left(y;p\right)}{\partial \eta^{2}}+Pr(R\hat{f}\left(\eta ;p\right)\frac{\partial \hat{\theta }\left(y;p\right)}{\partial \eta }+N_{b}\frac{\partial \hat{\theta }\left(y;p\right)}{\partial \eta }\frac{\partial \hat{\phi }\left(y;p\right)}{\partial \eta }+N_{t}{\left(\frac{\partial \hat{\theta }\left(y;p\right)}{\partial \eta }\right)}^{2}),$$ (46)

$$N_{G}\left(\hat{f}\left(\eta ;p\right),\hat{G}\left(\eta ;p\right)\right)=N_{2}\frac{\partial^{2}\hat{g}\left(y;p\right)}{\partial \eta^{2}}-N_{1}\left(2\hat{G}\left(\eta ;p\right)+\frac{\partial^{2}\hat{f}\left(\eta ;p\right)}{\partial \eta^{2}}\right)-N_{3}R(\frac{\partial \hat{f}\left(\eta ;p\right)}{\partial \eta }\hat{g}\left(\eta ;p\right)-\frac{\partial \hat{g}\left(\eta ;p\right)}{\partial \eta }\hat{f}\left(\eta ;p\right))+,$$ (47)

$$N_{\phi }\left(\hat{f}\left(\eta ;p\right),\hat{\theta }\left(\eta ;p\right),\hat{\phi }\left(\eta ;p\right)\right)=\frac{\partial^{2}\hat{\phi }\left(y;p\right)}{\partial y^{2}}+R.S\;c\;\hat{f}\left(y;p\right)\frac{\partial \hat{\phi }\left(y;p\right)}{\partial y}+\frac{N_{t}}{N_{b}}\frac{\partial^{2}\hat{\theta }\left(y;p\right)}{\partial y^{2}}.$$ (48)

When *p* varies from 0 to 1, we have

$$\begin{array}{r} \hat{f}\left(\eta ;0\right)=f_{0}\left(\eta \right),\hat{f}\left(\eta ;1\right)=f\left(\eta \right),\\ \hat{g}\left(\eta ;0\right)=g_{0}\left(\eta \right),\hat{g}\left(\eta ;1\right)=g\left(\eta \right),\\ \hat{\theta }\left(\eta ;0\right)=\theta_{0}\left(\eta \right),\hat{\theta }\left(\eta ;1\right)=\theta \left(\eta \right),\\ \hat{G}\left(\eta ;0\right)=G_{0}\left(\eta \right),\hat{G}\left(\eta ;1\right)=G\left(\eta \right),\\ \hat{\phi }\left(\eta ;0\right)=\phi_{0}\left(\eta \right),\hat{\phi }\left(\eta ;1\right)=\phi \left(\eta \right), \end{array}\}.$$ (49)

By means of Taylor's series

$$\begin{array}{l} f_{m}\left(\eta \right)=\frac{1}{m!}{\frac{\hat{f}\left(\eta ;p\right)}{\partial \eta^{m}}|}_{p=0},g_{m}\left(\eta \right)=\frac{1}{m!}{\frac{\hat{g}\left(\eta ;p\right)}{\partial \eta^{m}}|}_{p=0},G_{m}\left(\eta \right)=\frac{1}{m!}{\frac{\hat{G}\left(\eta ;p\right)}{\partial \eta^{m}}|}_{p=0},\\ \;\theta_{m}\left(\eta \right)=\frac{1}{m!}{\frac{\hat{\theta }\left(\eta ;p\right)}{\partial \eta^{m}}|}_{p=0},\phi_{m}\left(\eta \right)=\frac{1}{m!}{\frac{\hat{\phi }\left(y;p\right)}{\partial y^{m}}|}_{p=0}. \end{array}$$ (50)

The auxiliary converging parameters are chosen in such a way that the series (50) converges when *p* = 1. Thus we have

$$f\left(\eta \right)=f_{0}\left(\eta \right)+\sum_{m=1}^{\infty }f_{m}\left(\eta \right),$$ (51)

$$g\left(\eta \right)=g_{0}\left(\eta \right)+\sum_{m=1}^{\infty }g_{m}\left(\eta \right),$$ (52)

$$\theta \left(\eta \right)=\theta_{0}\left(\eta \right)+\sum_{m=1}^{\infty }\theta_{m}\left(\eta \right),$$ (53)

$$G\left(\eta \right)=G_{0}\left(\eta \right)+\sum_{m=1}^{\infty }G_{m}\left(\eta \right),$$ (54)

$$\phi \left(\eta \right)=\phi_{0}\left(\eta \right)+\sum_{m=1}^{\infty }\phi_{m}\left(\eta \right).$$ (55)

The resulting *mth* – *order* deformation equations are

$$L_{f}\left\{f_{m}\left(\eta \right)-\chi_{m-1}f_{m-1}\left(\eta \right)\right\}=c_{0}^{f}R_{m}^{f}\left(\eta \right),$$ (56)

$$L_{g}\left\{g_{m}\left(\eta \right)-\chi_{m-1}g_{m-1}\left(\eta \right)\right\}=c_{0}^{g}R_{m}^{g}\left(\eta \right),$$ (57)

$$L_{\theta }\left\{\theta_{m}\left(\eta \right)-\chi_{m-1}\theta_{m-1}\left(\eta \right)\right\}=c_{0}^{\theta }R_{m}^{\theta }\left(\eta \right),$$ (58)

$$L_{G}\left\{G_{m}\left(\eta \right)-\chi_{m-1}G_{m-1}\left(\eta \right)\right\}=c_{0}^{G}R_{m}^{G}\left(\eta \right),$$ (59)

$$L_{\phi }\left\{\phi_{m}\left(\eta \right)-\chi_{m-1}\phi_{m-1}\left(\eta \right)\right\}=c_{0}^{\phi }R_{m}^{\phi }\left(\eta \right),$$ (60)

$$f_{m}\left(0\right)=0,\;f_{m}\left(1\right)=0,\;f\prime {}_{m}\left(0\right)=0,\;f\prime {}_{m}\left(1\right)=0,\;G_{m}\left(0\right)=-nf\prime \prime {}_{m}\left(0\right),\;g_{m}\left(0\right)=0,\;g_{m}\left(1\right)=0,\;\theta_{m}\left(0\right)=0,\;\theta_{m}\left(1\right)=0,\;G_{m}\left(0\right)=nf\prime \prime {}_{m}\left(0\right),\;\phi_{m}\left(0\right)=0,\;\phi_{m}\left(1\right)=0,$$ (61)

with the following definitions

$$R_{m}^{f}\left(\eta \right)=\left(1+N_{1}\right)f^{iv}{}_{m-1}-R\left(\sum_{k=0}^{m-1}{f\prime }_{k}{f\prime \prime }_{m-1-k}-\sum_{k=0}^{m-1}f_{k}{f\prime \prime \prime }_{m-1-k}\right)+N_{1}g\prime \prime {}_{m-1}-Mf\prime \prime {}_{m-1}-2Kr{g\prime }_{m-1},$$ (62)

$$R_{m}^{g}\left(\eta \right)=\left(1+N_{1}\right){g\prime \prime }_{m-1}+R\left(\sum_{k=0}^{m-1}f_{k}{g\prime }_{m-1-k}-\sum_{k=0}^{m-1}g_{k}{f\prime }_{m-1-k}\right)-Mg_{m-1}+2Kr{f\prime }_{m-1},$$ (63)

$$R_{m}^{\theta }\left(\eta \right)={\theta \prime \prime }_{m-1}+\mathrm{Pr}\left(R\sum_{k=0}^{m-1}f_{k}{\theta \prime }_{m-1-k}+Nb\sum_{k=0}^{m-1}{\theta \prime }_{k}{\phi \prime }_{m-1-k}+Nt\sum_{k=0}^{m-1}{\theta \prime }_{k}{\theta \prime }_{m-1-k}\right),$$ (64)

$$R_{m}^{G}\left(\eta \right)=N_{2}{G\prime \prime }_{m-1}-N_{1}\left(2G_{m-1}+{f\prime \prime }_{m-1}\right)-N_{3}R(\sum_{k=0}^{m-1}{f\prime }_{k}G_{m-1-k}\sum_{k=0}^{m-1}{G\prime }_{k}f_{m-1-k}),$$ (65)

$$R_{m}^{\phi }\left(\eta \right)={\phi \prime \prime }_{m-1}+Sc.R\sum_{k=0}^{m-1}f_{k}{\phi \prime }_{m-1-k}+\frac{Nt}{Nb}{\theta \prime \prime }_{m-1},$$ (66)

in which

$$\chi_{m}=\{\begin{array}{c} 0,m\leq 1\\ 1,m>1 \end{array}.$$ (67)

The general solutions of Eqs (56)–(60) can be written as

$$f_{m}\left(\eta \right)=f_{m}^{*}\left(\eta \right)+C_{0}+C_{1}\eta +C_{2}\eta^{2}+C_{3}\eta^{3},$$ (68)

$$g_{m}\left(\eta \right)=g_{m}^{*}\left(\eta \right)+C_{4}+C_{5}\eta ,$$ (69)

$$\theta_{m}\left(\eta \right)=\theta_{m}^{*}\left(\eta \right)+C_{6}+C_{7}\eta ,$$ (70)

$$G_{m}\left(\eta \right)=G_{m}^{*}\left(\eta \right)+C_{8}+C_{9}\eta ,$$ (71)

$$\phi_{m}\left(\eta \right)=\phi_{m}^{*}\left(\eta \right)+C_{10}+C_{11}\eta ,$$ (72)

in which $f_{m}^{*}\left(y\right),g_{m}^{*}\left(y\right),\;\theta_{m}^{*}\left(y\right),G_{m}^{*}\left(y\right)$ and $\phi_{m}^{*}\left(y\right)$ are the particular solutions of the Eqs (68)–(72) Note that Eqs (56)–(60) can be solved by Mathematica one after the other in the order *m* = 1,2,3, …

#### Optimal convergence-control parameters

We know that the homotopic series solutions involve the non-zero auxiliary parameters $c_{0}^{f},c_{0}^{g},c_{0}^{\theta },c_{0}^{G}$ and $\;c_{0}^{\phi }$ which define the convergence-region of the Homotopy series solutions. To determine the optimal values of $c_{0}^{f},c_{0}^{g},c_{0}^{\theta },c_{0}^{G}$ and $c_{0}^{\phi }$ we utilize the concept of minimization by defining the average squared residual errors as introduced by [24].

$$E_{m}^{f}=\frac{1}{k+1}\sum_{j=0}^{k}\{N_{f}{\left({\sum_{i=0}^{m}\hat{f}\left(\eta \right),\sum_{i=0}^{m}\hat{g}\left(\eta \right),\sum_{i=0}^{m}\hat{G}\left(\eta \right))}_{\eta =j\delta \eta }\right\}}^{2}d\eta ,$$ (73)

$$E_{m}^{g}=\frac{1}{k+1}\sum_{j=0}^{k}{\left\{N_{g}{\left(\sum_{i=0}^{m}\hat{f}\left(\eta \right),\sum_{i=0}^{m}\hat{g}\left(\eta \right)\right)}_{\eta =j\delta \eta }\right\}}^{2}d\eta ,$$ (74)

$$E_{m}^{\theta }=\frac{1}{k+1}\sum_{j=0}^{k}{\left\{N_{\theta }{\left(\sum_{i=0}^{m}\hat{f}\left(\eta \right),\sum_{i=0}^{m}\hat{\theta }\left(\eta \right),\sum_{i=0}^{m}\hat{\phi }\left(\eta \right)\right)}_{\eta =j\delta \eta }\right\}}^{2}d\eta ,$$ (75)

$$E_{m}^{G}=\frac{1}{k+1}\sum_{j=0}^{k}{\left\{N_{G}{\left(\sum_{i=0}^{m}\hat{f}\left(\eta \right),\sum_{i=0}^{m}\hat{G}\left(\eta \right)\right)}_{\eta =j\delta \eta }\right\}}^{2}d\eta ,$$ (76)

$$E_{m}^{\phi }=\frac{1}{k+1}\sum_{j=0}^{k}{\left\{N_{\phi }{\left(\sum_{i=0}^{m}\hat{f}\left(\eta \right),\sum_{i=0}^{m}\hat{\phi }\left(\eta \right)\right)}_{\eta =j\delta \eta }\right\}}^{2}d\eta .$$ (77)

Following Liao [24]

$$E_{m}^{t}=E_{m}^{f}+E_{m}^{g}+E_{m}^{\theta }+E_{m}^{G}+E_{m}^{\phi },$$ (78)

where $E_{m}^{t}$ is total averaged squared residual error, *δη* = 0.5, *k* = 20. Total and individual averaged squared residual errors are computed using *N*_1_ = *N*_2_ = *N*_3_ = 0.10, *R* = 0.20, *n* = 0.50, *M* = 0.10 = *Kr* = *Nt* = *Nb* = *λ*, *Pr* = 1 = *Sc*. By means of computational software Mathematica we obtained the total and individual average squared residual errors at various order of iterations using highly efficient Mathematica package ***BVPh*2**. **0** which can be found at http://numericaltank.sjtu.edu.cn/BVPh2_0.htm. The basic idea is to **minimize** the total average squared residuals and determining the corresponding local optimal convergence control parameters. For this purpose, Tables 1 and 2 are prepared for the case of various optimal convergence control parameters. Table 1 gives the minimum value of total averaged squared residual error at several order of iterations while Table 2 is presented for the individual average squared residual error at different orders of approximations using the optimal values from Table 1 at *m* = 6. It is quite obvious from these two tables that the averaged squared residual errors and total averaged squared residual errors continue decreasing as we increase the order of approximation. So, Optimal Homotopy Analysis Method is an extremely effective tool in obtaining convergent series solutions for highly nonlinear systems of differential equations.

**Table 1:** Optimal convergence control parameters and total averaged squared residual errors using BVPh2. 0 when N_1_ = N_2_ = N_3_ = 0.10, R = 0.20, n = 0.50, M = 0.10 = Kr = Nt = Nb = λ, Pr = 1 = Sc.

*m*	$c_{0}^{f}$	$c_{0}^{g}$	$c_{0}^{\theta }$	$c_{0}^{G}$	$c_{0}^{\phi }$	$E_{m}^{t}$	CPU TIME[S]
2.0	−0.79	−0.74	−1.06	−7.47	−0.83	5.86 × 10^−3^	3.88
4.0	−0.87	−0.76	−1.10	−8.16	−0.89	4.78 × 10^−6^	116.0
6.0	−0.89	−0.88	−0.91	−8.28	−1.08	1.67 × 10^−9^	2453

**Table 2:** Individual Averaged squared residual errors using optimal values at *m* = 6 from Table 1.

*m*	$E_{m}^{f}$	$E_{m}^{g}$	$E_{m}^{\theta }$	$E_{m}^{G}$	$E_{m}^{\phi }$	CPUTIME[S]
4.0	1.04 × 10^−5^	1.17 × 10^−8^	3.50 × 10^−9^	6.18 × 10^−8^	1.30 × 10^−7^	2.12
6.0	1.59 × 10^−9^	1.05 × 10^−12^	1.13 × 10^−12^	3.80 × 10^−11^	3.96 × 10^−11^	3.98
10	7.40 × 10^−16^	2.38 × 10^−20^	1.46 × 10^−19^	2.39 × 10^−17^	4.57 × 10^−18^	8.69
14	4.85 × 10^−22^	3.44 × 10^−28^	1.91 × 10^−26^	1.58 × 10^−23^	4.31 × 10^−25^	15.28

### 2. Numerical Solution

Despite OHAM, the governing system of Eqs (10)–(14) is also solved numerically using midpoint integration as a basic scheme and Richardson extrapolation as an enhancement scheme with highly efficient computational software Maple as used by several other authors [26–30] This scheme works by transforming the governing system of nonlinear higher order differential equations into a system of first order ordinary differential equations which are then solved using iterative schemes through midpoint integration as it belongs to the class of higher order Runge-Kutta methods. A mesh size of Δ*h* = 0.001 was set for a convergence criterion of 10^−6^ in our computations. Our computed numerical results are in very good agreement with analytical results obtained by optimal homotopy analysis method. We are thus confident that our applied numerical algorithm is up to the mark.

## Results and Discussion

The aim here is to discuss the behavior of velocity, temperature, micro rotation and concentration profiles against emerging physical parameters in our flow problem. Figs 2–25 are plotted for this purpose. Figs 2–7 are plotted to determine the influence of coupling parameter *N*_1,_ spin gradient viscosity parameter *N*_2_, rotation parameter *Kr*, Hartman number *M*, porosity parameter *λ* and Reynolds number *R* on velocity profile *f*′(*η*). From Figs 2 and 3 we observe that the influence of coupling parameter *N*_1_ and spin gradient viscosity parameter *N*_2_ on the velocity profile *f*′(*η*) is similar, i.e. it initially decreases near the lower stretching plate and from the center of the plates towards the upper plate the behavior is reversed.

**Fig 2:**
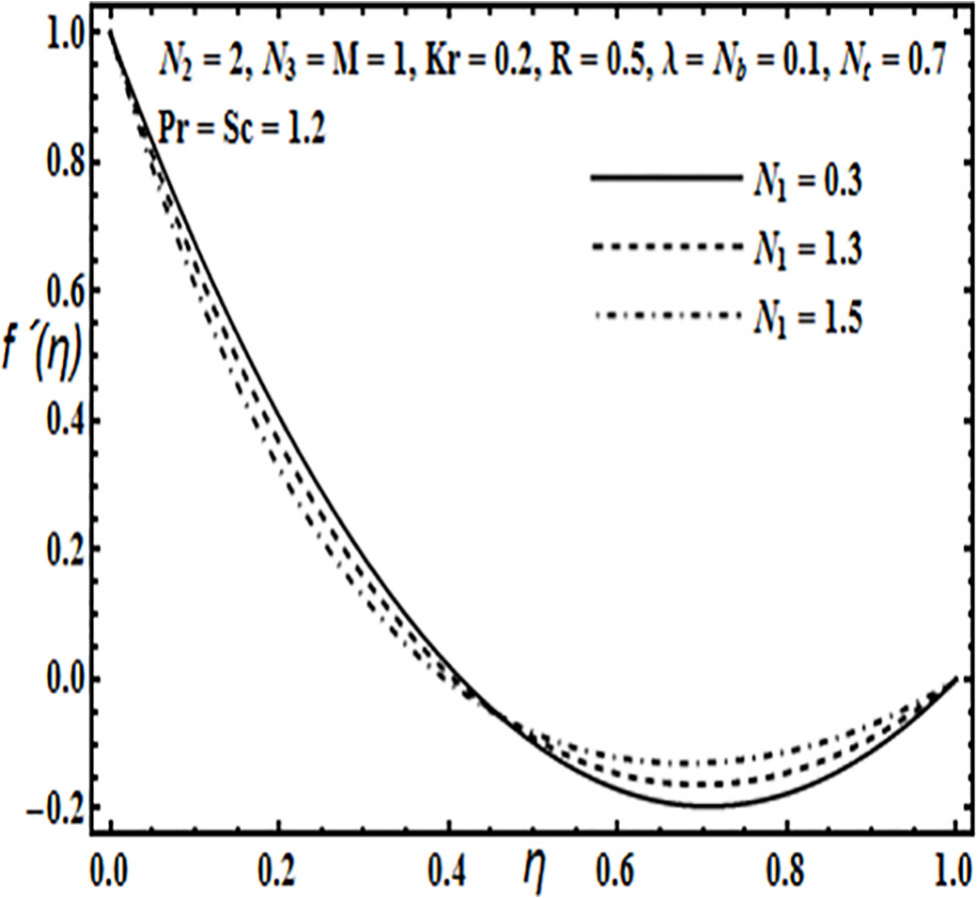
Effect of *N*_1_ on *f*′(*η*).

**Fig 3:**
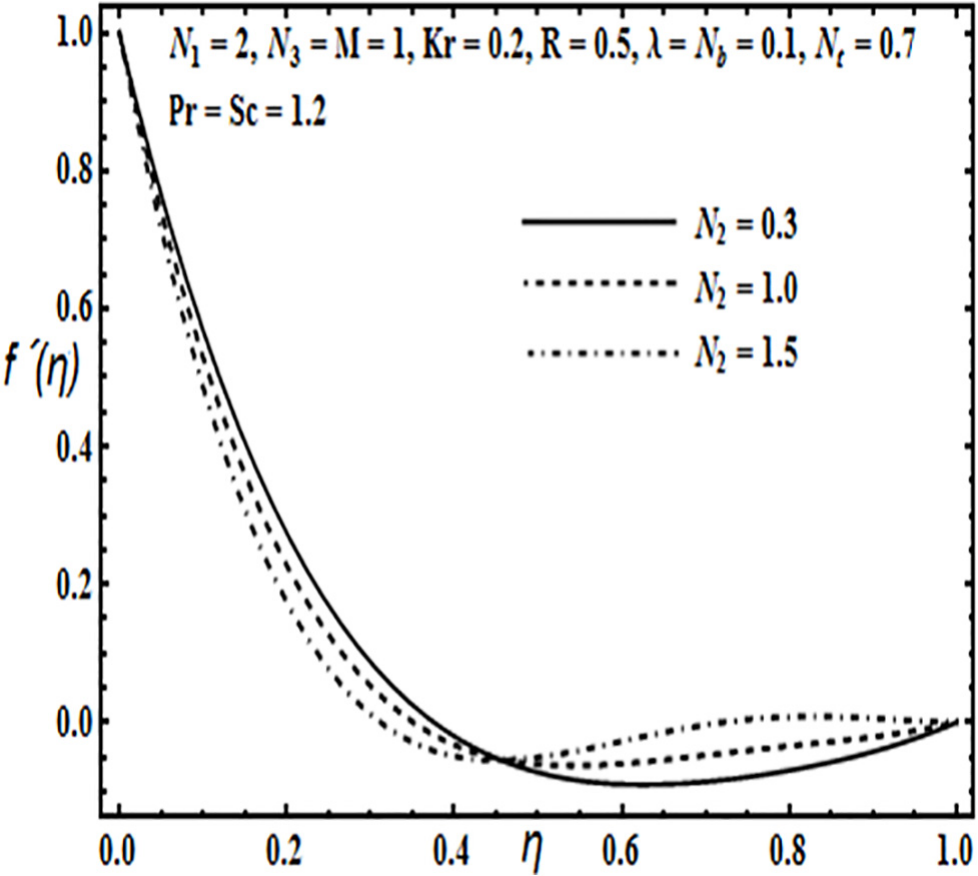
Effect of *N*_2_ on *f*′(*η*).

**Fig 4:**
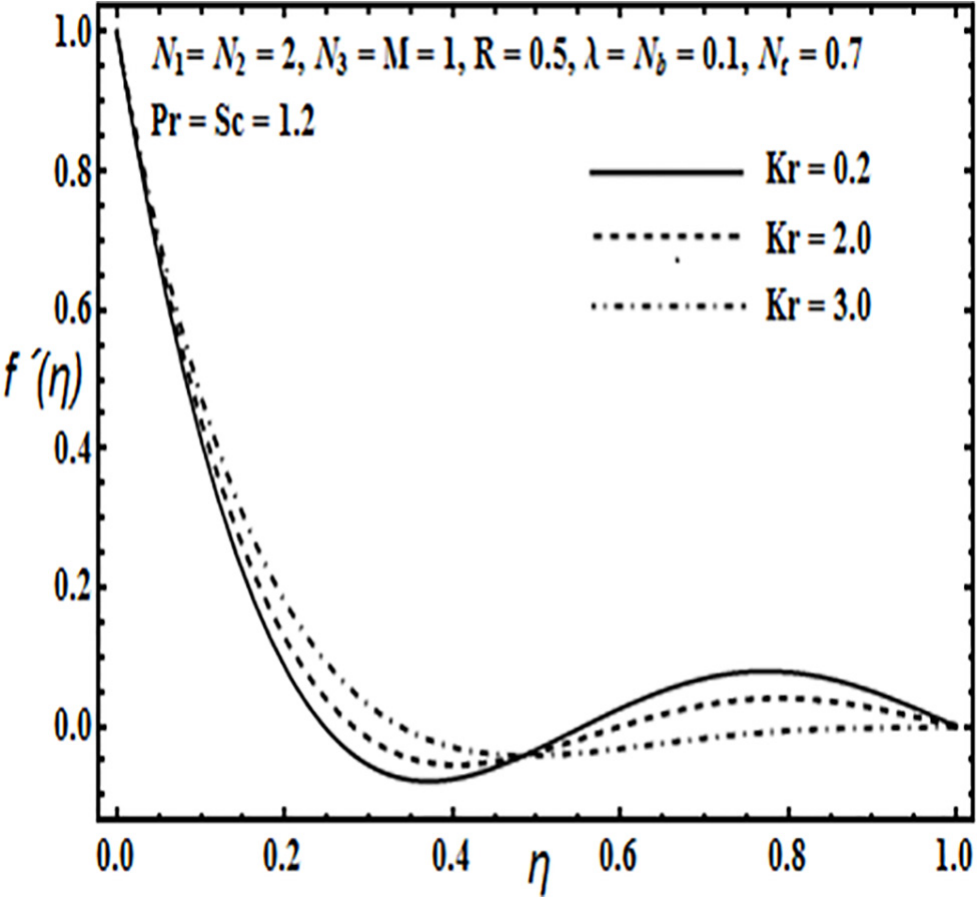
Effect of *Kr* on *f*′(*η*).

**Fig 5:**
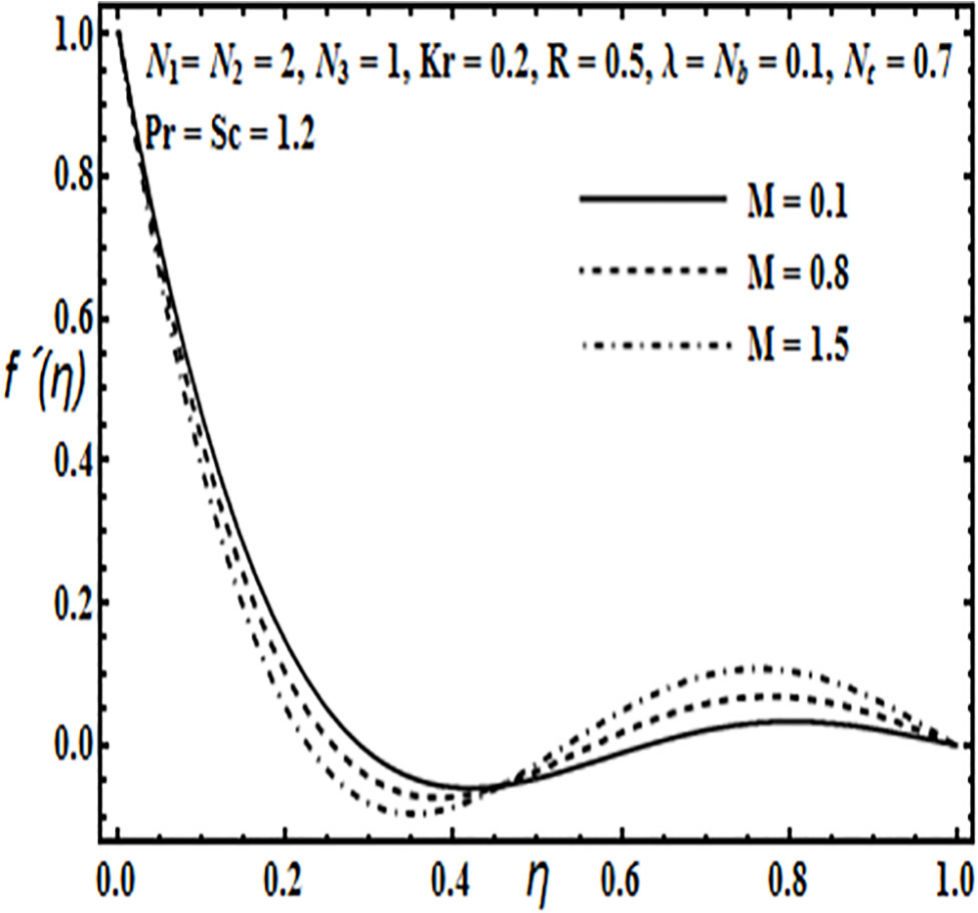
Effect of *M* on *f*′(*η*).

**Fig 6:**
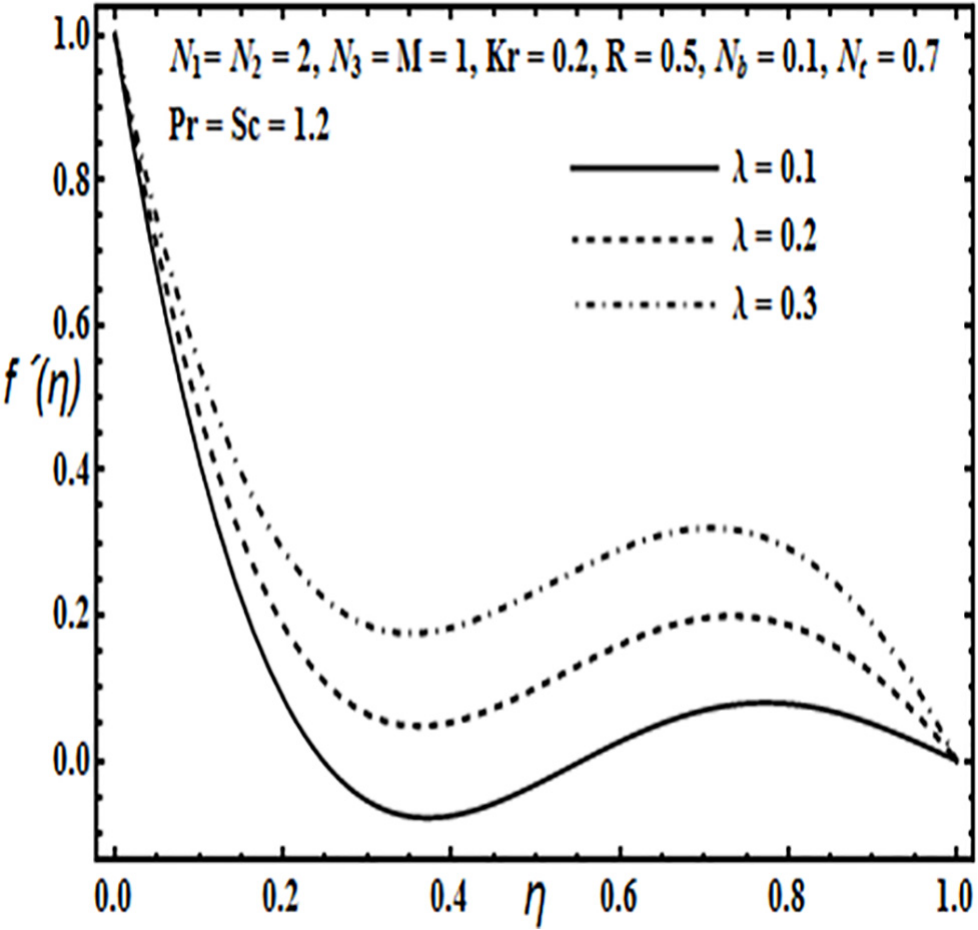
Effect of *λ* on *f*′(*η*).

**Fig 7:**
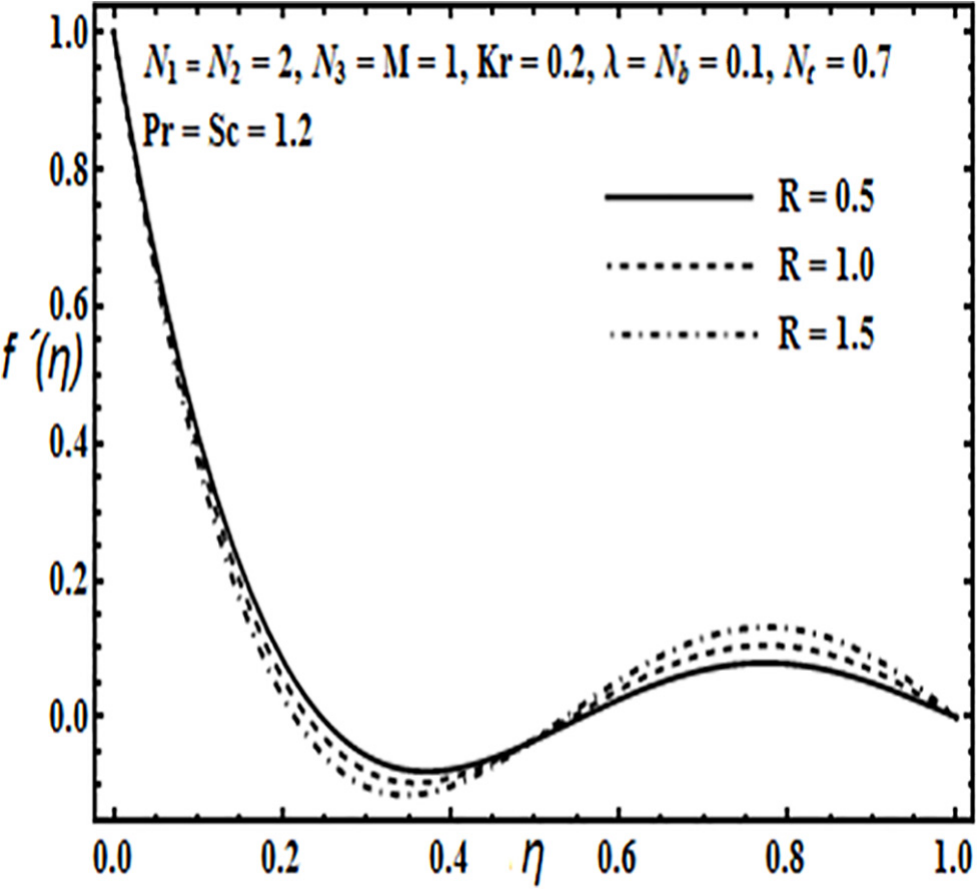
Effect of *R* on *f*′(*η*).

**Fig 8:**
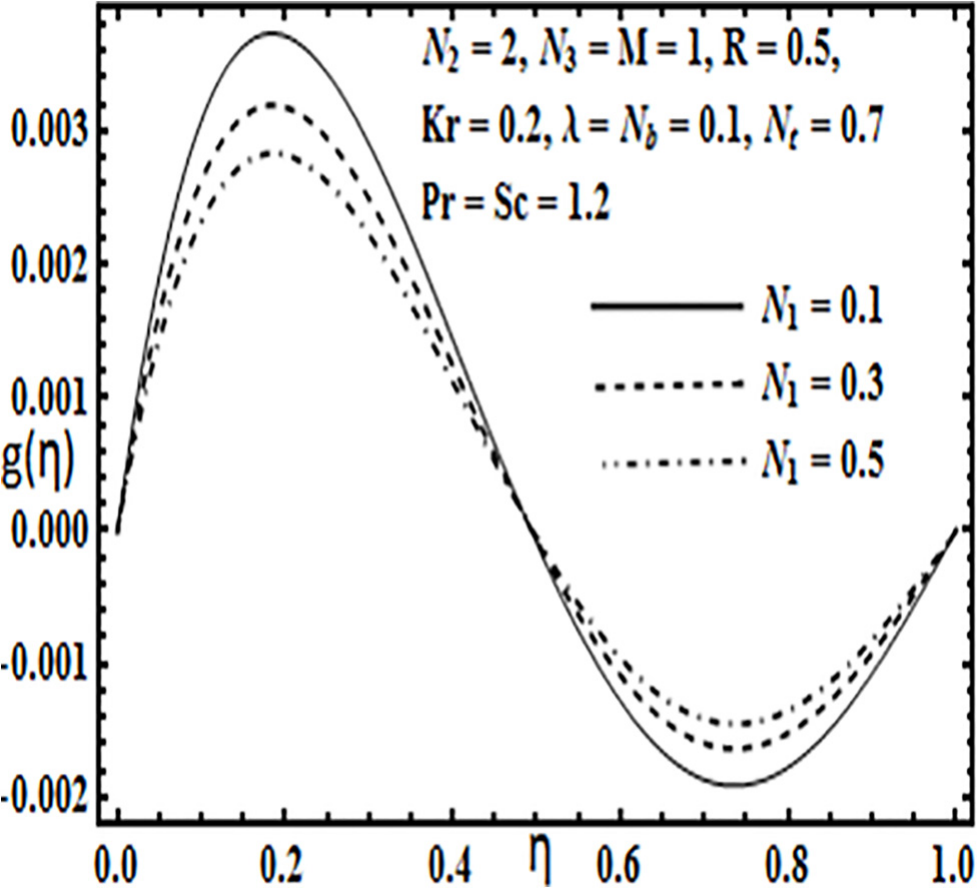
Effect of *N*_1_ on *g*(*η*).

**Fig 9:**
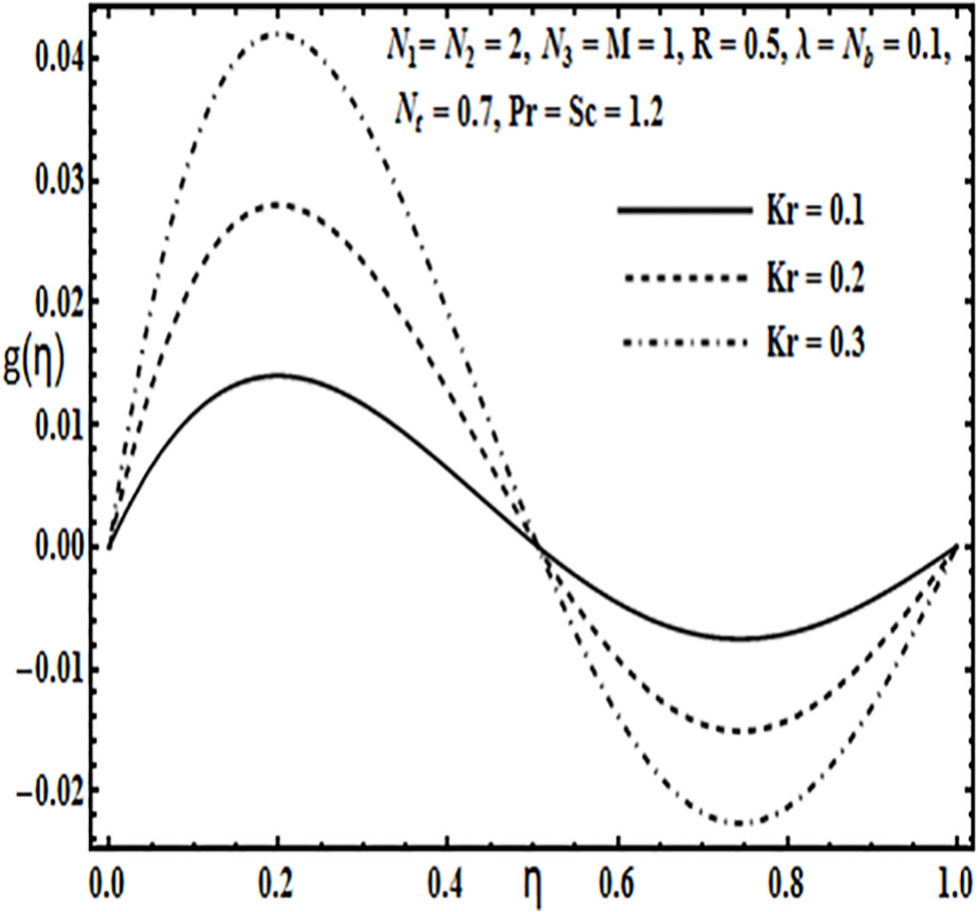
Effect of *Kr* on *g*(*η*).

**Fig 10:**
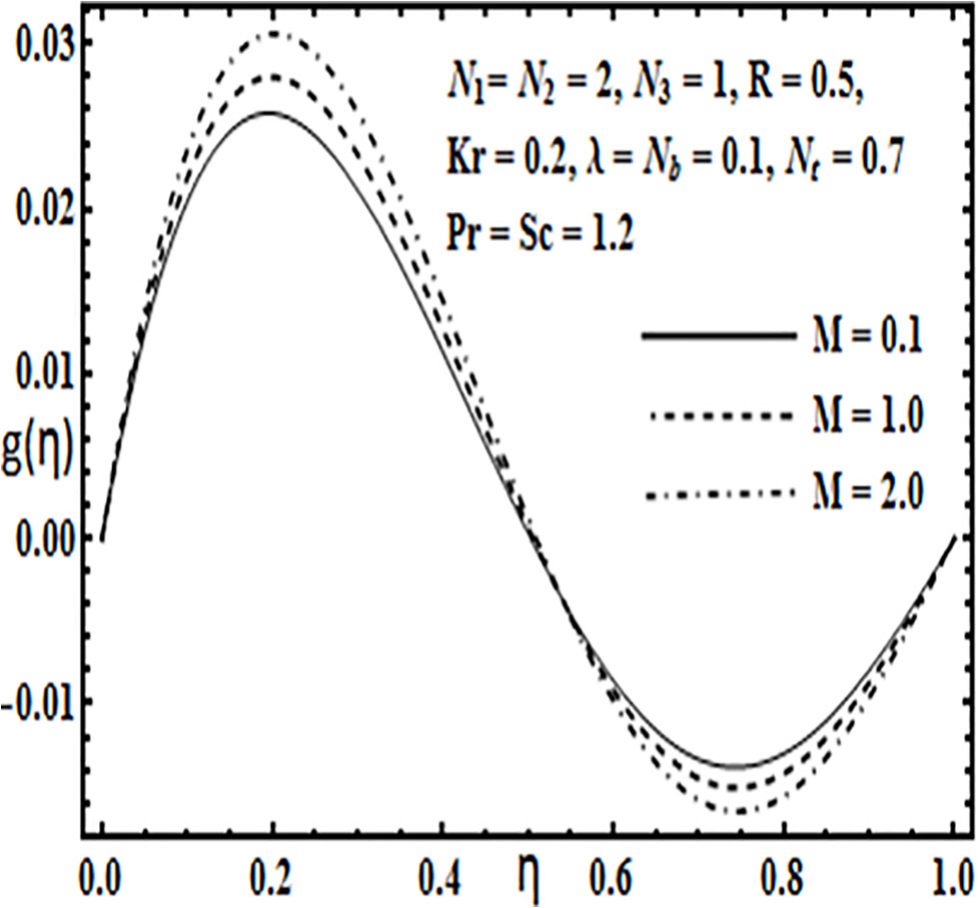
Effect of *M* on *g*(*η*).

**Fig 11:**
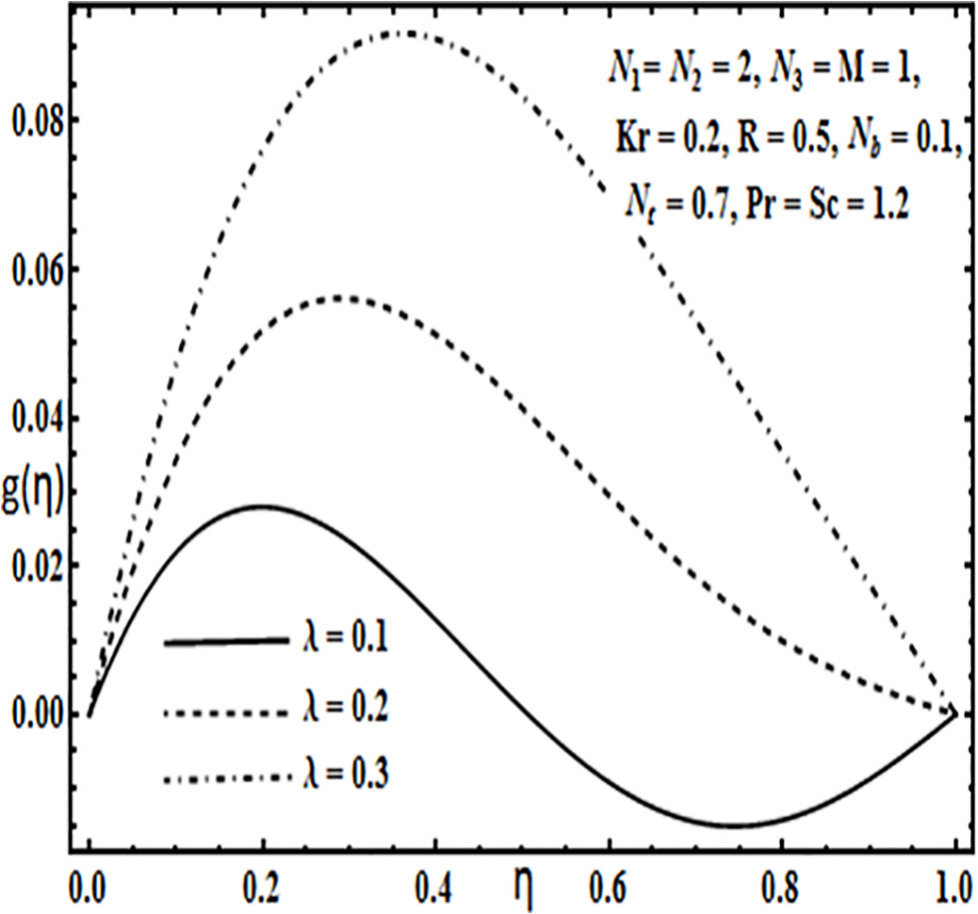
Effect of *λ* on *g*(*η*).

**Fig 12:**
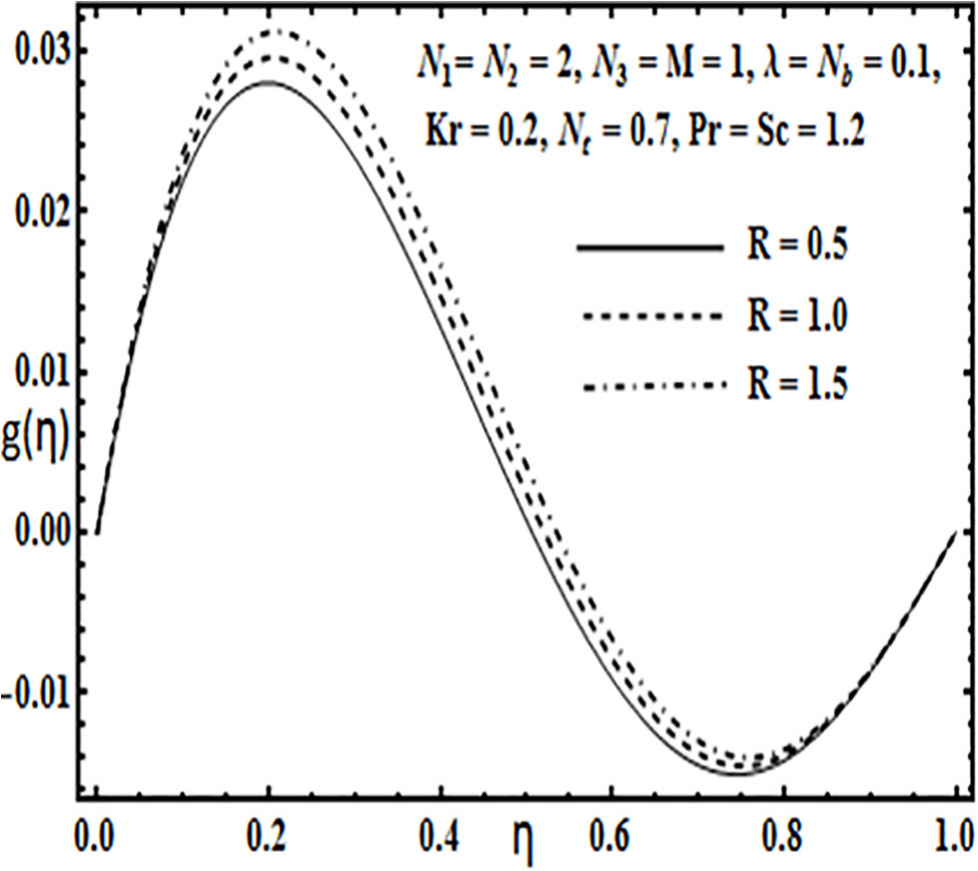
Effect of *R* on *g*(*η*).

**Fig 13:**
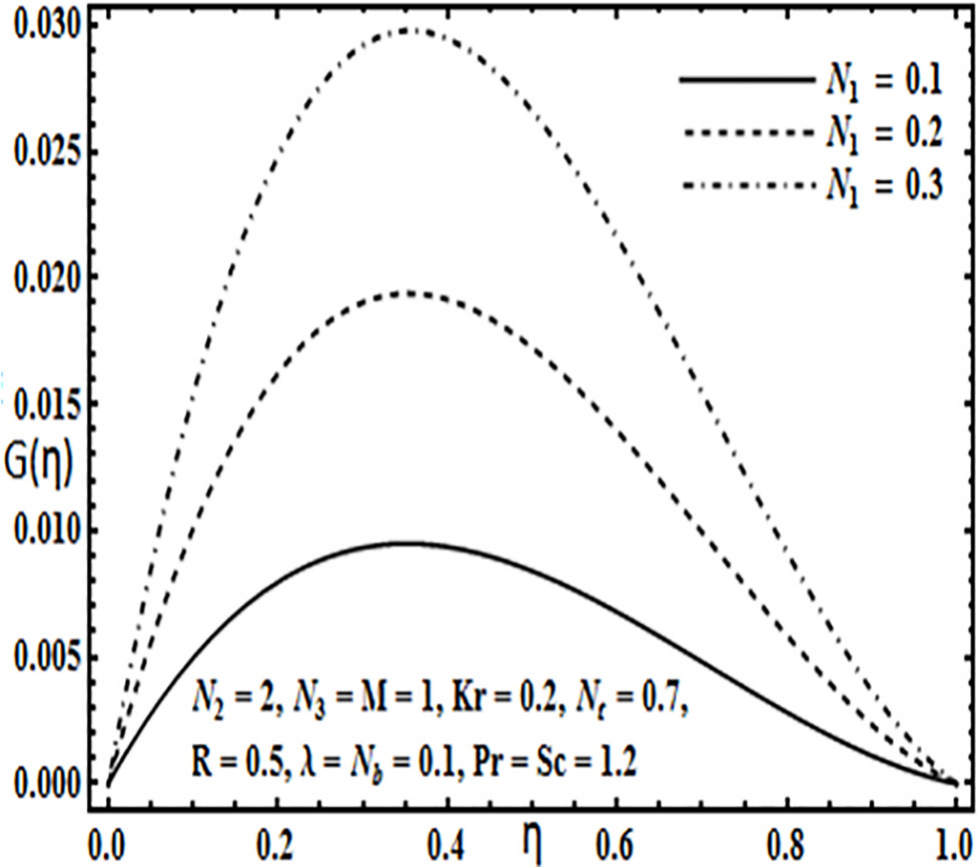
Effect of *N*_1_ on *G*(*η*).

**Fig 14:**
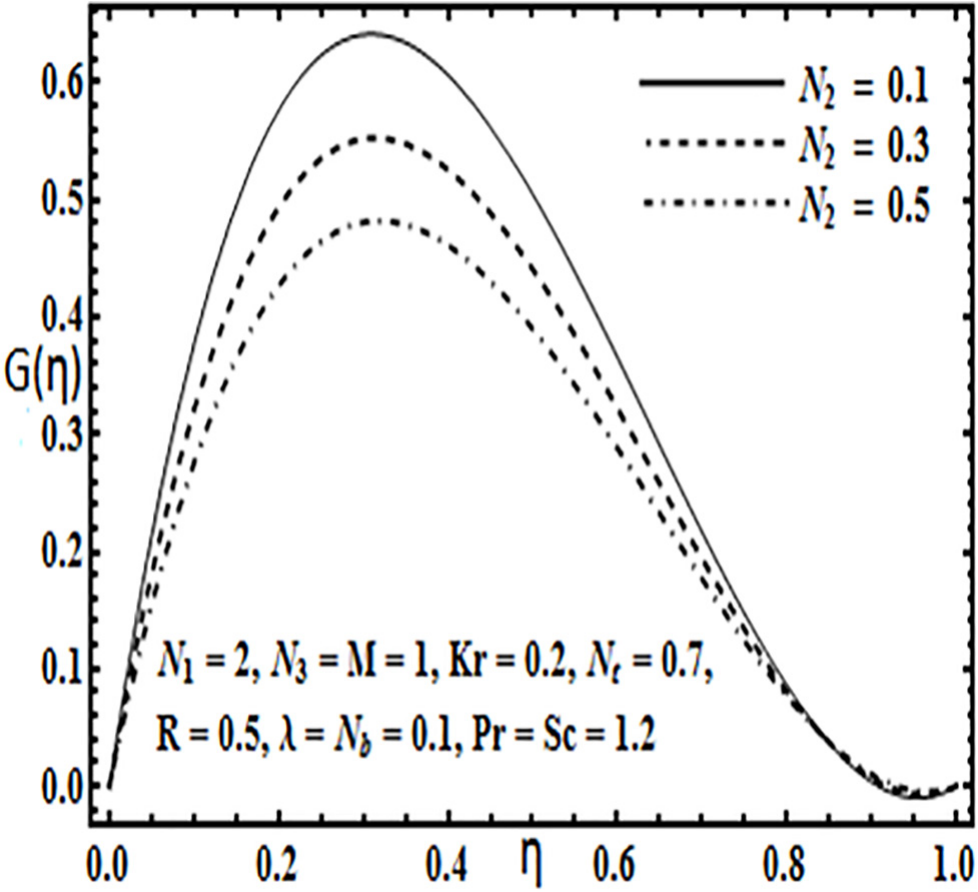
Effect of *N*_2_ on *G*(*η*).

**Fig 15:**
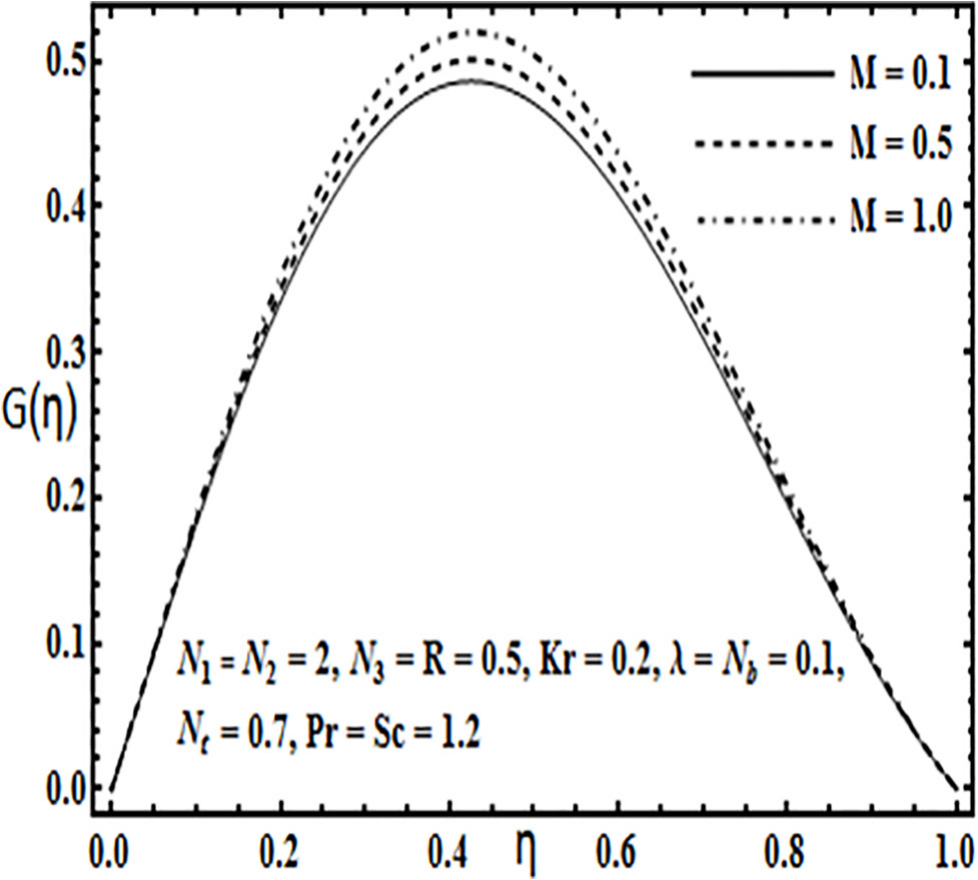
Effect of *M* on *G*(*η*).

**Fig 16:**
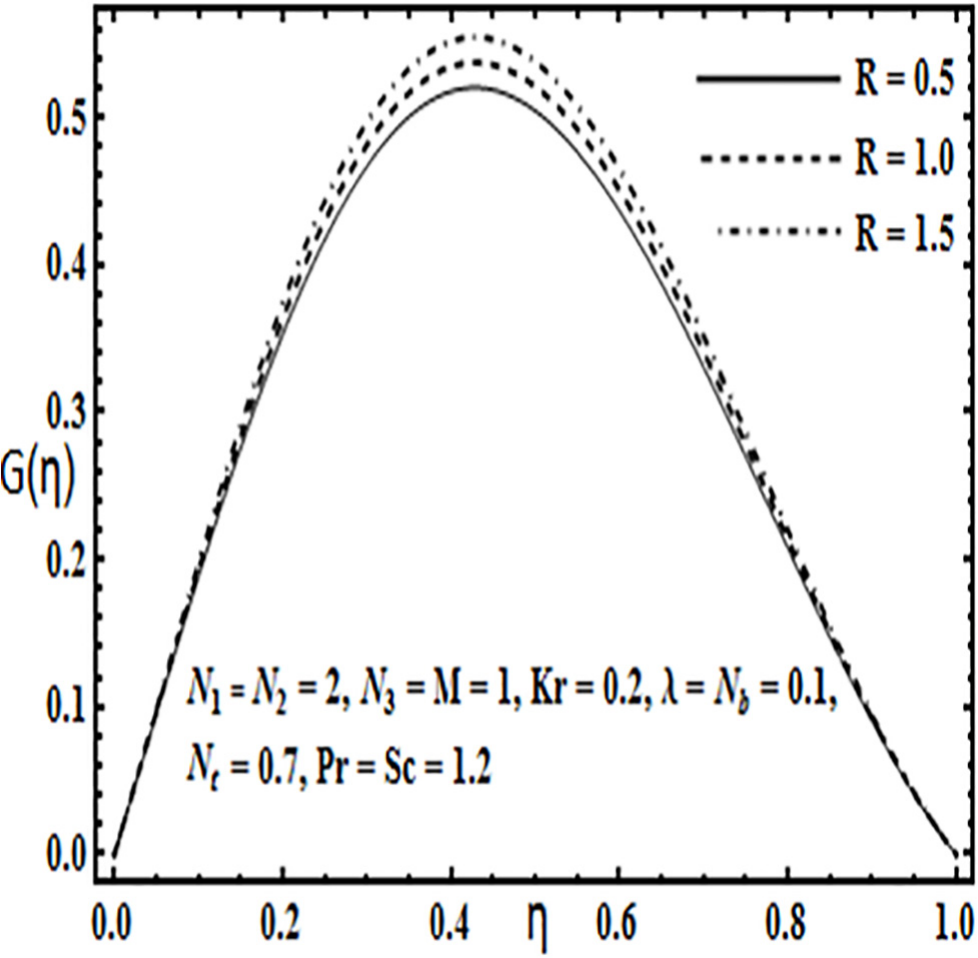
Effect of *R* on *G*(*η*).

**Fig 17:**
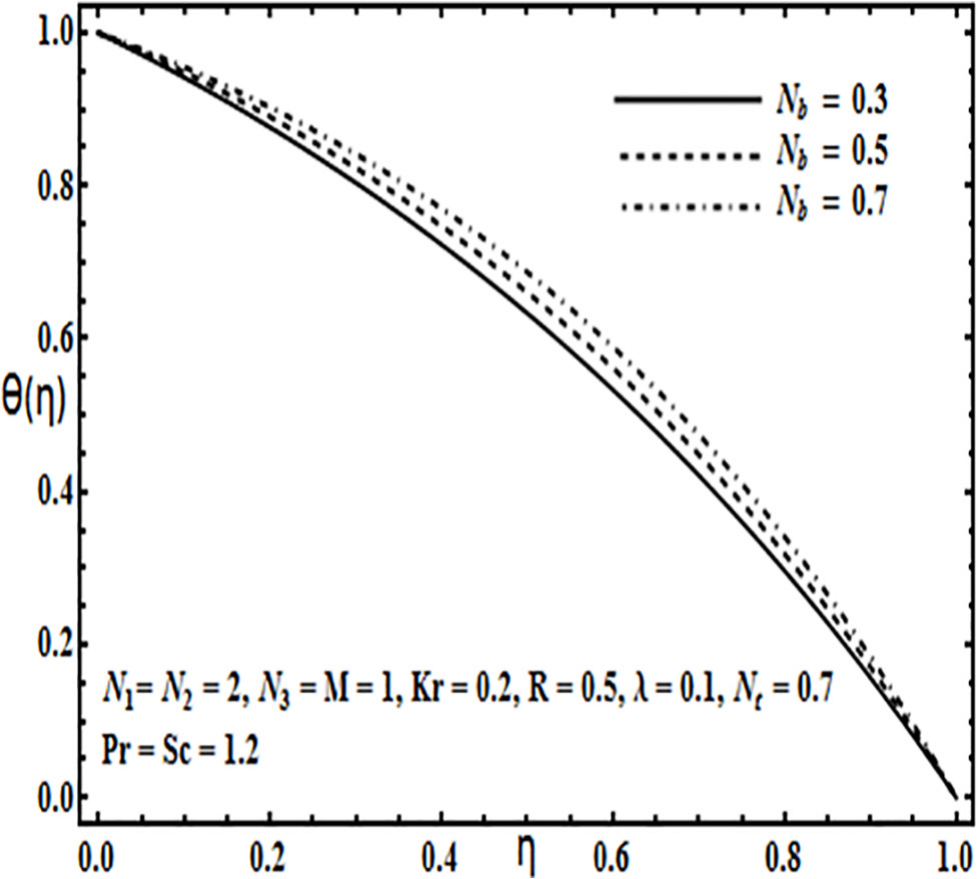
Effect of *N*_*b*_ on *θ*(*η*).

**Fig 18:**
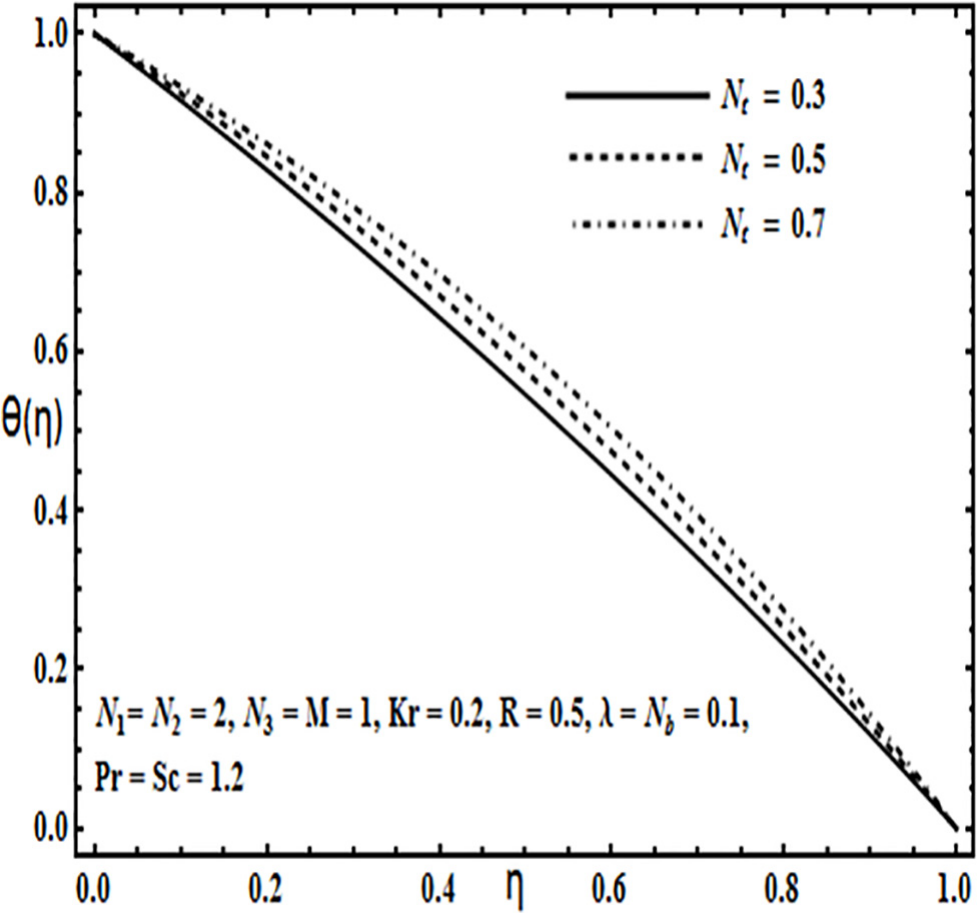
Effect of *N*_*t*_ on *θ*(*η*).

**Fig 19:**
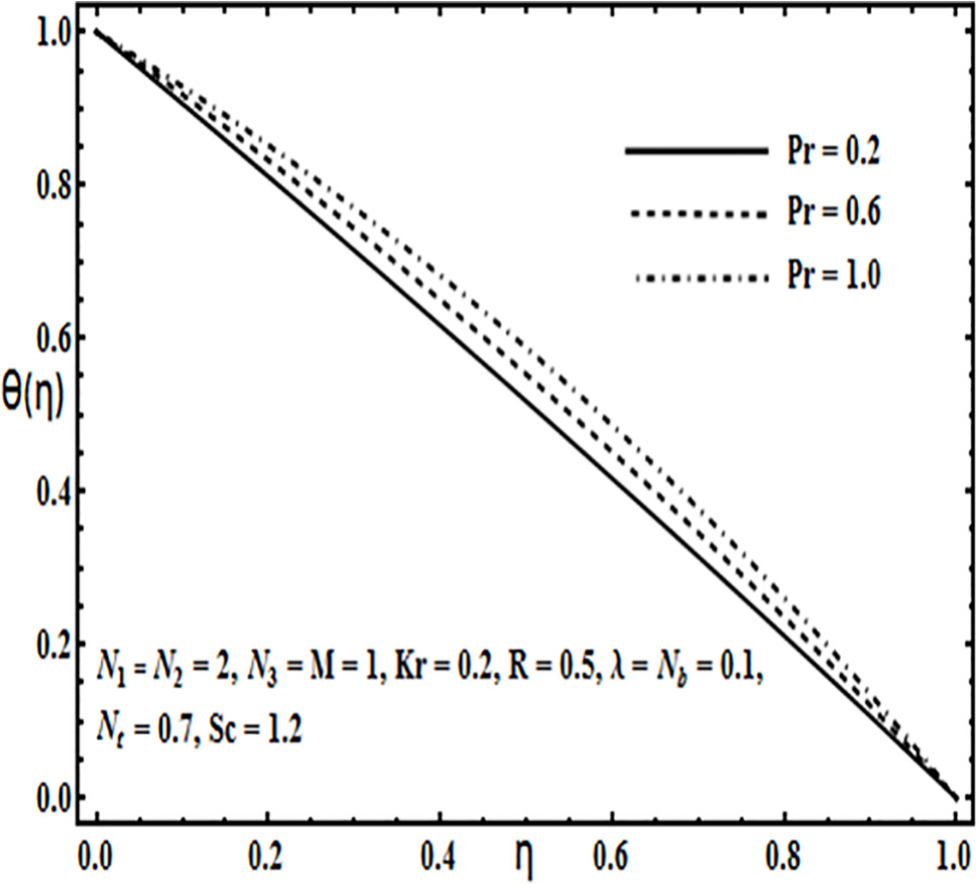
Effect of *Pr* on *θ*(*η*).

**Fig 20:**
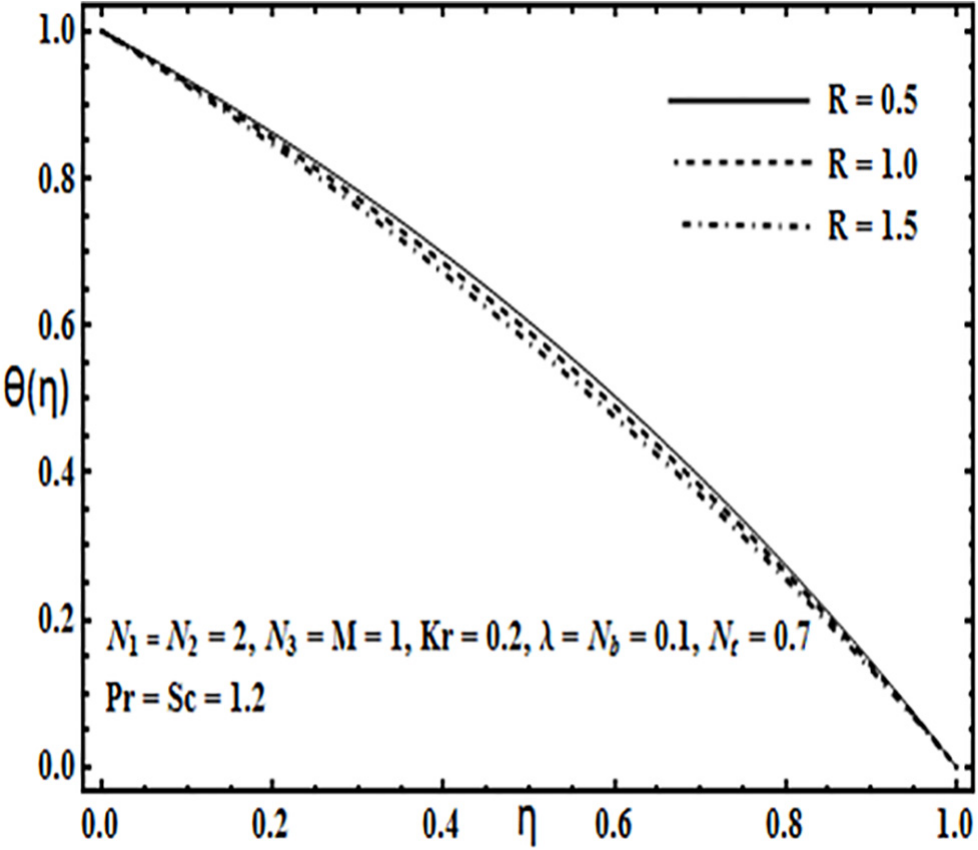
Effect of *R* on *θ*(*η*).

**Fig 21:**
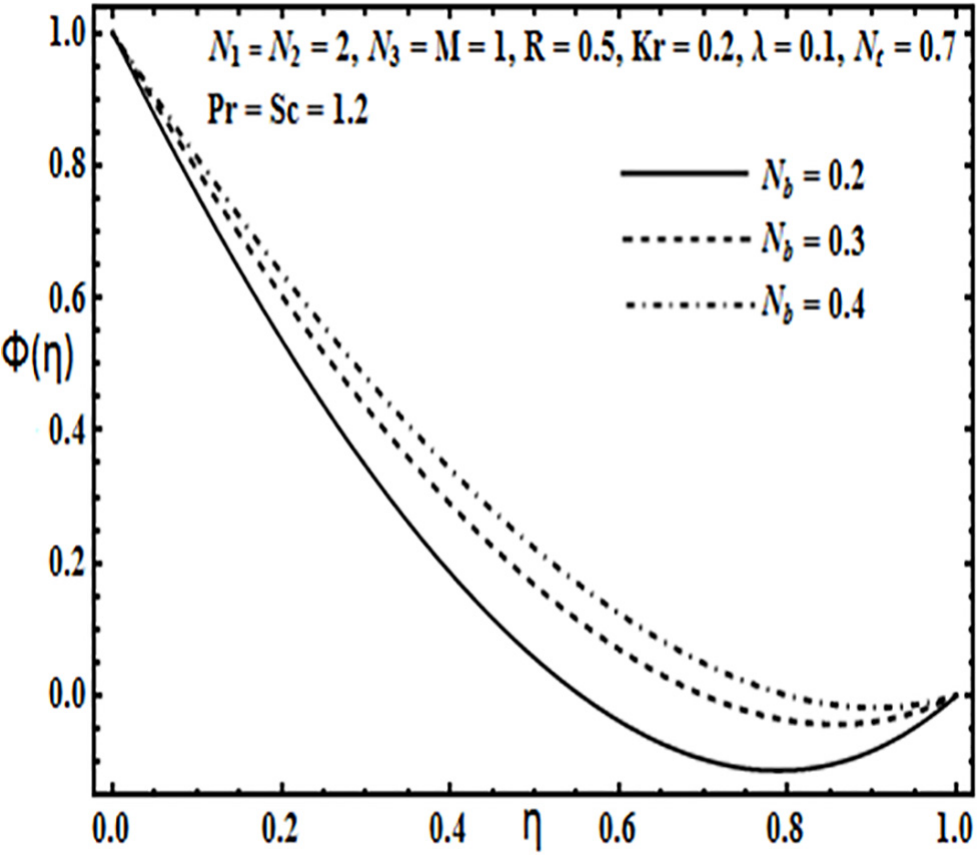
Effect of *N*_*b*_ on *ϕ*(*η*).

**Fig 22:**
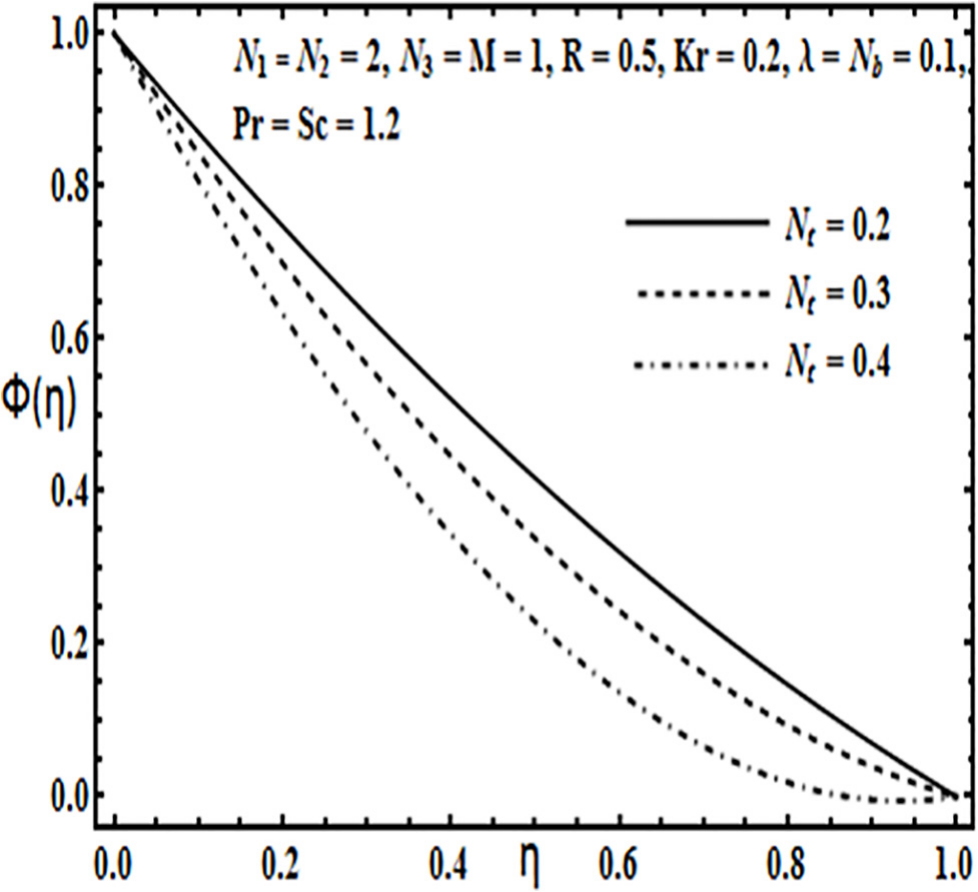
Effect of *N*_*t*_ on *ϕ*(*η*).

**Fig 23:**
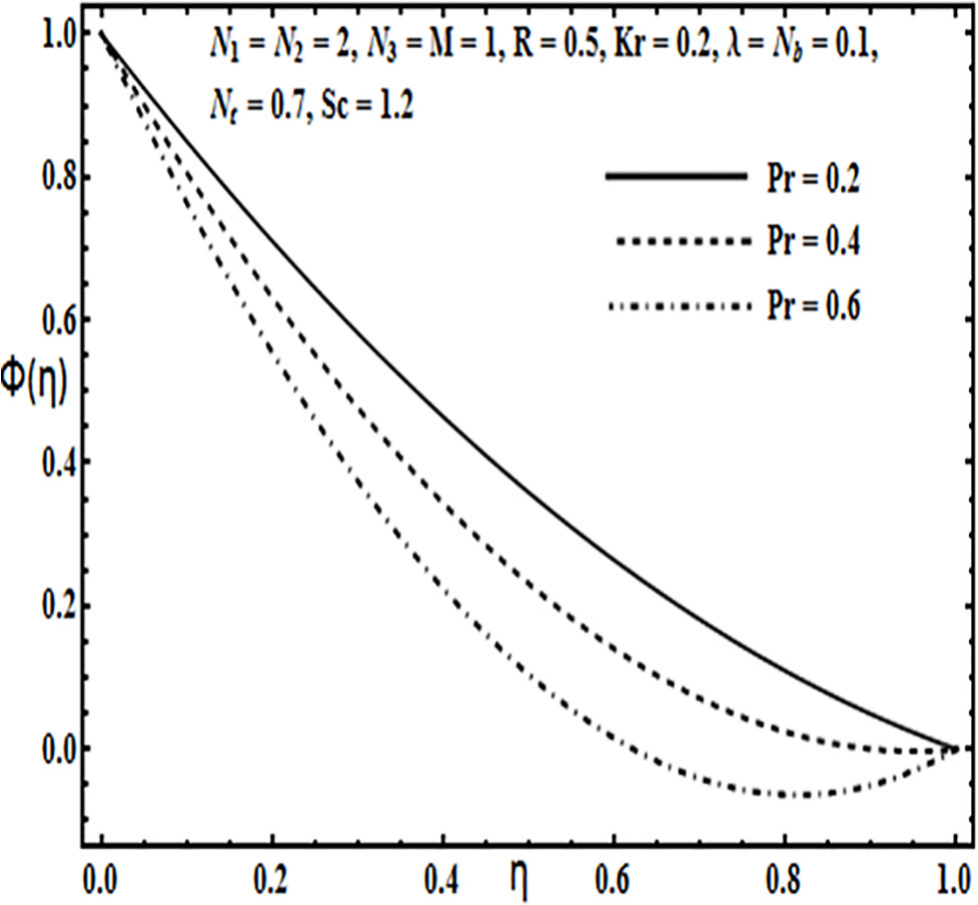
Effect of *Pr* on *ϕ*(*η*).

**Fig 24:**
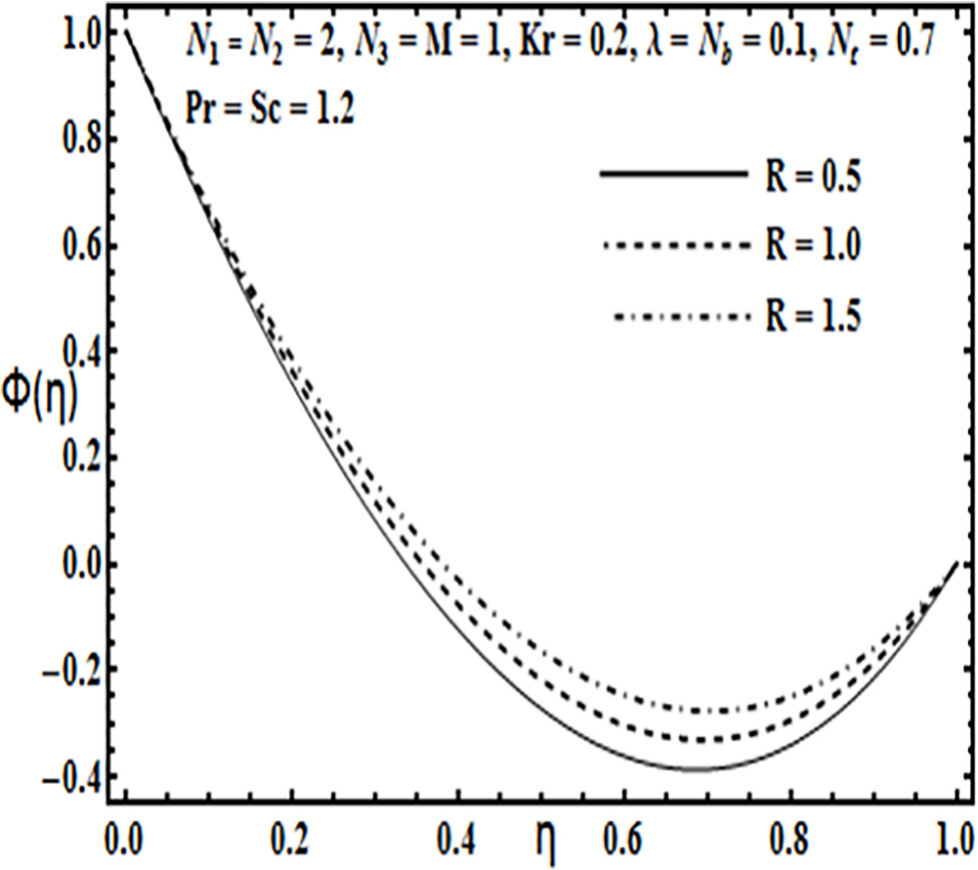
Effect of *R* on *ϕ*(*η*).

**Fig 25:**
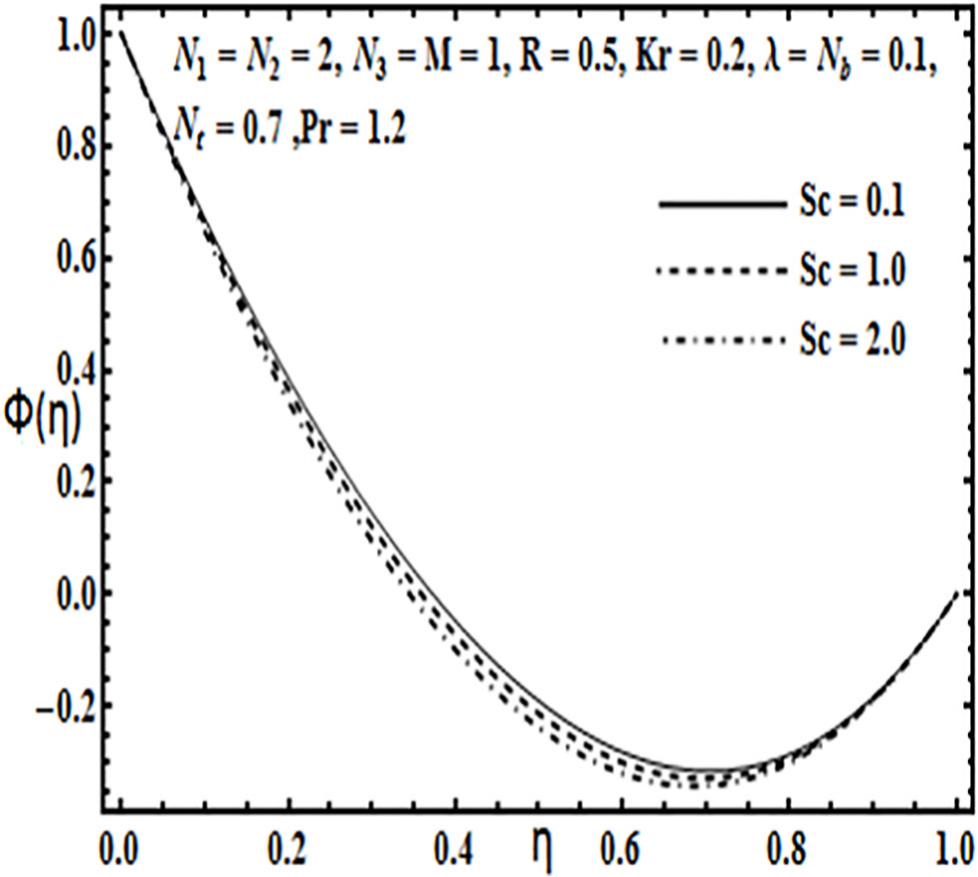
Effect of *Sc* on *ϕ*(*η*).

On the other hand, from Figs 4 and 5 velocity profile *f*′(*η*) increases towards the main stream with an increase in rotation parameter *Kr* Hartman number *M* and then it tends to decrease towards the upper plate.

The effects of porosity parameter *λ* and Reynolds number *R* are revealed through Figs 6 and 7. From Fig 6 we observe that increasing the porosity parameter *λ* results in an increase in the velocity profile *f*′(*η*) from lower towards the upper plate. From Fig 7 velocity profile *f*′(*η*) decreases near the lower plate but increases towards the upper plate.

The impact of embedding physical parameters such as coupling parameter *N*_1_, rotation parameter *Kr*, Hartman number *M*, porosity parameter *λ* and Reynolds number *R* on transverse velocity *g*(*η*) is displayed through Figs 8–12. Figs 8 and 9 depict that coupling parameter *N*_1_ and rotation parameter *Kr* have opposite influence on transverse velocity *g*(*η*). Similarly from Fig 10 increasing the magnetic field through Hartman number *M* increases the transverse velocity *g*(*η*) near the lower plate but contrary behavior is observed near the upper plate. It is quite evident from Figs 11 and 12 that increasing the porosity parameter *λ* and Reynolds number *R* have a positive influence on the transverse velocity *g*(*η*).

Variation in the micro rotation profile *G*(*η*) against various parameters such as coupling parameter *N*_1_, spin gradient viscosity parameter *N*_2_, Hartman number *M* and Reynolds number *R* are presented through Figs 13–16. From Figs 13 and 14 the effects of coupling parameter *N*_1_ and spin gradient viscosity parameter *N*_2_ on the micro rotation profile *G*(*η*) are quite opposite to each other. From Fig 13 increasing the coupling parameter *N*_1_ leads to an increase in the micro rotation profile *G*(*η*) while it decreases with an increase in spin gradient viscosity parameter *N*_2_ (See Fig 14). Influence of Hartman number *M* and Reynolds number *R* on the micro rotation profile *G*(*η*) is found to be similar from Figs 15 and 16. Both these parameters enhance the micro ration profile *G*(*η*) between the parallel plates.

The impact of physical parameters such as Brownian motion parameter *N*_*b*_, thermophoresis parameter *N*_*t*_, Prandtl number *Pr* and Reynolds number *R* on the temperature profile *θ*(*η*) is presented through Figs 17–20. From Figs 17 and 18 temperature profile *θ*(*η*) increases significantly with an increase in Brownian motion *N*_*b*_ as well as thermophoresis parameter *N*_*t*_. Figs 19 and 20 illustrate effects of Prandtl number *Pr* and Reynolds number *R* on the temperature profile *θ*(*η*). Temperature profile *θ*(*η*) increases with an increase in Prandtl number *Pr*(*Fig* 19), while it decreases with increasing Reynolds number *R* (*Fig* 20).

The effect of physical parameters such as Brownian motion parameter *N*_*b*_, thermophoresis parameter *N*_*t*_, Prandtl number *Pr*, Reynolds number *R* and Schmidt number *Sc* on the concentration profile *φ*(*η*) is presented through Figs 21–25. Figs 21 and 22 indicate that Brownian motion parameter *N*_*b*_ increases while thermophoresis parameter *N*_*t*_ decreases the concentration profile *φ*(*η*) between the plates. From Figs 23 and 24 concentration profile *φ*(*η*) decreases with an increase in Prandtl number *Pr* while it increases with an increase in Reynolds number *R*. Finally Fig 25 shows that for large values of Schmidt number *Sc*, concentration profile *φ*(*η*) decreases between the parallel plates.

Physical quantities of interest such as skin friction co-efficient, Nusselt and Sherwood number are computed through Tables 3–5. Table 3 examines the influence of embedding parameters such as *N*_1_, *N*_3_, *M*, *Kr* and *R* on skin friction coefficient. All these parameters have a positive influence on skin friction at the lower plate but it is interesting to mention here that this increasing behavior is more prominent for the case of strong concentration i.e. *n* = 0.0 when compared to the case of weak concentration *n* = 0.5. The impact of coupling parameter *N*_1_, Brownian motion parameter *N*_*b*_, thermophoresis parameter *N*_*t*_, Reynolds number *R* and Schmidt number *Sc* on the Nusselt number is depicted through Table 4. For higher values of coupling parameter *N*_1_, Nusselt number –*θ*′(0) tends to increase while it decreases with an increase in, Brownian motion parameter *N*_*b*_, thermophoresis parameter *N*_*t*_. Similarly, it is also observed that with an increase in Reynolds number *R*, Nusselt number –*θ*′(0) also increases while it decreases with an increase in Schmidt number *Sc* for strong *n* = 0.0 as well as weak concentration *n* = 0.5. Finally Table 5 depicts that Sherwood number –*ϕ*′(0) responds in an opposite manner against Brownian motion parameter *N*_*b*_, thermophoresis parameter *N*_*t*_. Moreover local mass flux –*ϕ*′(0) decreases with Reynolds number *R* and it increases with an increase in Schmidt number *Sc* for strong *n* = 0.0 as well as weak concentration *n* = 0.5.

**Table 3:** Numerical values of skin friction at the wall when *N*_2_ = 1.0.

					C_*f*_ = (1 + *N*_1_)*f* "(0) + *N*_1_ *G*(0)			
					*n* = 0.0 (Strong Concentration)		*n* = 0.50 (Weak Concentration)	
*N*_1_	*N*_3_	M	Kr	*R*	*Numerical*	*Optimal Ham*	*Numerical*	*Optimal Ham*
0.0	0.1	0.1	0.1	0.5	−3.46067	−3.46067	−3.46067	−3.46067
0.1					−3.79947	−3.79947	−3.62896	−3.62896
0.5					−5.13213	−5.13213	−4.31894	−4.31894
0.1	0.0	0.1	0.1	0.5	−3.79947	−3.79947	−3.62881	−3.62881
	0.3				−3.79947	−3.79947	−3.62924	−3.62924
	0.5				−3.79947	−3.79947	−3.62953	−3.62953
0.1	0.1	0.0	0.1	0.5	−3.78723	−3.78723	−3.61727	−3.61727
		0.5			−3.84813	−3.84813	−3.67540	−3.67540
		1.0			−3.90829	−3.90829	−3.73284	−3.73284
0.1	0.1	0.1	0.0	0.5	−3.79942	−3.79942	−3.62890	−3.62890
			0.5		−3.80078	−3.80078	−3.63020	−3.63020
			1.0		−3.80486	−3.80486	−3.63410	−3.63410
0.1	0.1	0.1	0.1	0.5	−3.79947	−3.79947	−3.62896	−3.62896
				1.0	−3.84769	−3.84769	−3.67514	−3.67514
				1.5	−3.89581	−3.89581	−3.72123	−3.72123

**Table 4:** Numerical values of heat flux at the wall when N_2_ = 1.0, N_3_ = 0.1, λ = 0.1, M = 0.1, Kr = 0.1, Pr = 1.

					*Nu* = −*θ* ′(0)			
					*n* = 0.0 (Strong Concentration)		*n* = 0.50 (Weak Concentration)	
*N*_1_	*N*_*b*_	*N*_*t*_	R	Sc	*Numerical*	*Optimal Ham*	*Numerical*	*Optimal Ham*
0.0	0.1	0.1	0.5	1.0	0.92042	0.92042	0.92042	0.92042
0.1					0.92044	0.92044	0.92043	0.92043
0.3					0.92049	0.92049	0.92043	0.92043
0.1	0.5	0.1	0.5	1.0	0.71075	0.71075	0.71074	0.71074
	0.8				0.57831	0.57831	0.57831	0.57831
	1.2				0.43240	0.43240	0.43240	0.43240
0.1	0.1	0.5	0.5	1.0	0.71285	0.71285	0.71284	0.71284
		0.8			0.58125	0.58125	0.58124	0.58124
		1.2			0.43578	0.43578	0.43575	0.43575
0.1	0.1	0.1	0.5	1.0	0.92044	0.92044	0.92043	0.92043
			1.0		0.95655	0.95655	0.95653	0.95653
			1.5		0.99309	0.99309	0.99306	0.99306
0.1	0.1	0.1	0.5	0.5	0.92078	0.92078	0.92077	0.92077
				1.0	0.92044	0.92044	0.92043	0.92043
				1.5	0.92010	0.92010	0.92009	0.92009

**Table 5:** Numerical values of Mass flux at the wall when N_1_ = N_2_ = 1.0, N_3_ = 0.1, λ = 0.1, M = 0.1, Kr = 0.1, Pr = 1.2.

				*Sh* = −*φ* ′(0)			
				*n* = 0.0 (Strong Concentration)		*n* = 0.50 (Weak Concentration)	
*N*_*b*_	*N*_*t*_	R	Sc	*Numerical*	*Optimal Ham*	*Numerical*	*Optimal Ham*
0.5	0.1	0.5	1.0	1.09115	1.09115	1.09058	1.09058
0.8				1.08604	1.08604	1.08571	1.08571
1.2				1.08064	1.08064	1.08030	1.08030
0.1	0.2	0.5	1.0	1.30437	1.30437	1.30476	1.30476
	0.5			2.47999	2.47999	2.48122	2.48122
	0.8			4.41360	4.41360	4.41532	4.41532
0.1	0.1	0.5	1.0	1.11270	1.11270	1.11274	1.11274
		1.0		1.10926	1.10926	1.10935	1.10935
		1.5		1.10493	1.10493	1.10509	1.10509
0.1	0.1	0.5	0.5	1.09558	1.09558	1.09580	1.09580
			1.0	1.11270	1.11270	1.11274	1.11274
			1.5	1.12995	1.12995	1.12980	1.12980

## Concluding Remarks

Hydromagnetic flow of micropolar nanofluid between two horizontal parallel plates in a rotating system has been investigated numerically as well as analytically using optimal HAM. The core outcomes of the study can be enlisted as

- The effects of coupling parameter *N*_1_ and Hartman number *M* on velocity profile *f*′(*η*) are found to be opposite compared to viscosity parameter *N*_2_ and rotation parameter *Kr*.
- Influence of magnetic field *M* and coupling parameter *N*_1_ on transverse velocity *g*(*η*) are opposing each other. Moreover it is observed that increasing the porosity parameter *λ* leads to an increase in the transverse velocity *g*(*η*).
- It is observed that temperature profile *θ*(*η*) enhances for large values of Prandtl number *Pr*, thermophoresis parameter *Nt* and Brownian motion parameter *Nb* for the case of strong concentration (*n* = 0).
- Micro rotation profile *G*(*η*) raises with coupling parameter *N*_1,_ magnetic field parameter *M* and Reynolds number *R* for strong concentration i.e. when *n* = 0.
- Increasing the thermophoresis parameter *Nt* and Brownian motion parameter *Nb* results in a contrary behavior on concentration profile *ϕ*(*η*).
- Skin friction at the lower plate tends to increase with magnetic field parameter *M*, rotation parameter *Kr* and Reynolds number *R*. This behavior is found to be more prominent for the case of strong concentration i.e. (*n* = 0) compared to the weak concentration (*n* = 0.5).
- Heat flux –*θ*′(0) at the lower plate drops with Increasing thermophoresis parameter *Nt* and Brownian motion parameter *Nb* while on the other hand mass flux –*ϕ*′(0) responds in an opposite manner against these two parameters.
